# Ensilicated tetanus antigen retains immunogenicity: *in vivo* study and time-resolved SAXS characterization

**DOI:** 10.1038/s41598-020-65876-3

**Published:** 2020-06-08

**Authors:** A. Doekhie, R. Dattani, Y-C. Chen, Y. Yang, A. Smith, A. P. Silve, F. Koumanov, S. A. Wells, K. J. Edler, K. J. Marchbank, J. M. H. van den Elsen, A. Sartbaeva

**Affiliations:** 1 Department of Chemistry, University of Bath, Claverton Down, Bath, BA2 7AY United Kingdom; 2 European Synchrotron Research Facility, ESRF, 71 avenue des Martyrs, CS 40220, 38043 Grenoble, Cedex 9 France; 3 Transnational and Clinical Research Institute, Newcastle University, Medical School, Newcastle upon Tyne, NE2 4HH United Kingdom; 4 Diamond Light Source, Diamond Light Source Ltd, Harwell Campus, Didcot, OX11 0DE United Kingdom; 5 Department for Health, University of Bath, Claverton Down, Bath, BA2 7AY United Kingdom; 6 Department of Biology and Biochemistry, University of Bath, Claverton Down, Bath, BA2 7AY United Kingdom; 7 Department of Chemical Engineering, University of Bath, Claverton Down, Bath, BA2 7AY United Kingdom; 8 Present Address: Department of Biochemistry, University of Cambridge, CB2 0QR Cambridge, UK; 9 Present Address: College of Pharmacy, Purdu Univeristy Heine Pharmacy Building, West Lafayette, IN 47907-2091 United States

**Keywords:** Materials chemistry, Physical chemistry, Surface chemistry, Biochemistry, Immunology

## Abstract

Our recently developed ensilication approach can physically stabilize proteins in silica without use of a pre-formed particle matrix. Stabilisation is done by tailor fitting individual proteins with a silica coat using a modified sol-gel process. Biopharmaceuticals, e.g. liquid-formulated vaccines with adjuvants, frequently have poor thermal stability; heating and/or freezing impairs their potency. As a result, there is an increase in the prevalence of vaccine-preventable diseases in low-income countries even when there are means to combat them. One of the root causes lies in the problematic vaccine ‘cold chain’ distribution. We believe that ensilication can improve vaccine availability by enabling transportation without refrigeration. Here, we show that ensilication stabilizes tetanus toxin C fragment (TTCF), a component of the tetanus toxoid present in the diphtheria, tetanus and pertussis (DTP) vaccine. Experimental *in vivo* immunization data show that the ensilicated material can be stored, transported at ambient temperatures, and even heat-treated without compromising the immunogenic properties of TTCF. To further our understanding of the ensilication process and its protective effect on proteins, we have also studied the formation of TTCF-silica nanoparticles via time-resolved Small Angle X-ray Scattering (SAXS). Our results reveal ensilication to be a staged diffusion-limited cluster aggregation (DLCA) type reaction. An early stage (tens of seconds) in which individual proteins are coated with silica is followed by a subsequent stage (several minutes) in which the protein-containing silica nanoparticles aggregate into larger clusters. Our results suggest that we could utilize this technology for vaccines, therapeutics or other biopharmaceuticals that are not compatible with lyophilization.

## Introduction

Biopharmaceuticals are biologically derived active compounds intended for therapeutic or diagnostic usage. Examples include monoclonal antibodies, vaccines, cells and blood components. Many of these are proteins and not stable *ex vivo*. They must be stabilised to increase their shelf-life. The main approach in stabilisation of biopharmaceuticals is regulating temperature. Maintaining low (2–8 °C) temperatures retains native states of these compounds. Many protein-based biopharmaceuticals are vulnerable not only to heating but also to freezing^1–3^, which can disrupt protein structure and lead to denaturation on thawing.

Within cold chain transportation^1,2,4–7^, from manufacturing to endpoint destination, temperature control has proven challenging. Fluctuations in temperature, heating/freezing^1^, can affect biopharmaceuticals resulting in aggregation and protein unfolding, leading to loss of potency. This is a serious issue in global public health^8–10^ as it is a major hindrance to achieving universal childhood vaccination worldwide. Many alternative approaches to render biopharmaceuticals more thermally stable for storage and transport have been proposed; for example the use of PLA (poly-lactic-acid) or PGLA (poly-c-glycolic-lactic acid)^11^, sugar glass^12^ and alginate/chitosan mixtures^13^.

Our previous research describes a new alternative to stabilise biopharmaceuticals, ensilication, which can be performed at ambient-to-low temperatures (4–25 °C) at neutral pH using solution-gelation (sol-gel) technology^14^ based upon the condensation and polymerisation of monomeric species^15^. This eventually forms a particle as product which, in contact with other particles, forms gels. We use a silica precursor, tetraethyl orthosilicate (TEOS) hydrolysed to form orthosilicic acid^16,17^. This is a central Si atom tetrahedrally coordinated by four hydroxyl (OH-) groups and capable of rapid polymerisation to form silica under appropriate conditions. Individual proteins can thus be coated with a form-fitting silica layer, nucleated at positively charged residues such as lysine and arginine on the protein surface. The protein-loaded silica powder that results is resilient against fluctuations in temperature.

In a recent study^18^, our group has shown that ensilication can be successfully applied to the tuberculosis antigen 85b, a candidate TB vaccine component, and to Ag85b conjugated with the *Staphylococcus aureus*-protein based adjuvant Sbi. Ensilication successfully protects these proteins from the loss of structure and immunogenic function that the unprotected proteins suffer when exposed to elevated temperatures or to long-term storage without refrigeration.

TTCF^19^ is a ~52 kilodalton (kDa) fragment of the full tetanus neurotoxin (TeNT) and is a potent immunogen. The DTP vaccine contains tetanus toxoid, the inactivated form of TeNT, and has been shown to be susceptible to problems in cold chain transportation. This is one of the contributory factors to the 16% lower vaccination coverage for DTP^10^ in countries with poor infrastructure, and results in higher prevalence of disease. Using TTCF as a model, we demonstrate that ensilication could be a solution to overcome the challenges of biopharmaceutical ‘cold chain’ transportation.

## Experimental

### Expression of recombinant TTCF in BL21(DE3) *E. coli*, ensilication and release

Expression of recombinant TTCF was performed in BL21(DE3) *E. coli* using the pET-16b-TTCF HisTag vector^19^. Protein expression was induced using IPTG and purified using immobilised metal affinity chromatography using a nickel binding HisTrap column. The eluted peak fraction was dialysed extensively against 50 mM Tris at pH 7.

Purity of purified TTCF was assessed before ensilication using SDS-PAGE (Fig. S1). The theoretical molecular weight of TTCF is 53545.5 Da and extinction coefficient is 85845 L mol^−1^ cm^−1^. Using these two parameters, concentration of the protein was determined via UV absorbance reading at 280 nm. Unless otherwise stated, purified TTCF was dialysed against 50 mM Tris pH 7.0 and stored at −20 °C until further use. The integrity of the TTCF was checked regularly before use to certify the functional integrity of the protein.

Ensilication for the preparation of a long-term storable product^14^ was performed as follows. Pre-hydrolysed TEOS was prepared by mixing 1:1 volumes of ultrapure H_2_O and TEOS. Acid-catalysed hydrolysis was initiated by the addition of 1:500 (v/v) (32%) HCl. To 15 ml of 1 mg/ml purified TTCF in 50 mM Tris pH 7.0, pre-hydrolysed TEOS was added in 1:50 (v/v) ratio with stirring at 100 rpm. Ensilication was monitored for 15 min and the product was vacuum filtered and dried for 24 hr at room temperature (RT, 20 °C). The final powder was collected and stored at RT (20 °C) for 1 month. The ensilicated powder was separated into two parts, one for heat treatment (proof-of-principle) and another without.

Release of ensilicated TTCF was performed by adding 10 mg of product to 5 ml of 50 mM Tris pH 7 and adding 5 ml of acidified NaF at pH 3. The powder was dissolved by placing the sample on a rotator, 60 rpm, for 1 hr at RT (20 °C) until the solution was clear.

### Circular Dichroism

Far-UV CD spectra between 260–185 nm were recorded at 20 °C for native, heated native, released and heat-treated released TTCF using a Chirascan CD spectrometer (Applied Photophysics, UK). The proteins were beforehand dialysed against sodium phosphate buffer at pH 7 and measured using a quartz cuvette with a 1 mm path length. Sample concentrations were determined after dialysis using the commercial BCA protein assay and were between 0.1 and 0.3 mg/ml. 5 scans were recorded for each sample with a bandwidth of 2 nm, step of 1 nm and time-per-point of 2 seconds. Scans were averaged and normalised by conversion of machine units (in millidegrees) to delta epsilon (∆ε) in M^−1^ cm^−1^.

### *in vivo* animal study

Ensilicated material for *in vivo* experiments were produced at the University of Bath. The *in vivo* trial was carried out at Newcastle University. Samples were stored for 1 month under ambient conditions before transport. The ensilicated powder was transported between the two facilities using commercial airline transport without use of cooling equipment. Half of the ensilicated TTCF was heat-treated at 80 °C for 2 hours, a regime which is denaturing to the unprotected protein.

TTCF after release from ensilication, using dilute fluoride solution^14^, was dialysed in 50 mM Tris-HCl pH 7.0 using a 10k MWCO Slide-A-Lyzer dialysis cassette (ThermoFisher, UK), before injection into mice. A total of 20 mice (Charles River lab.) in groups of 5 were used in a 48 day immunisation protocol. Groups were assigned as follows: native TTCF (+ve control); native TTCF + heated (−ve control), TTCF ensilicated then released^14^, TTCF ensilicated + heated then released. Following pre-immunisation bleed, mice received an intraperitoneal injection of 5 µg/dose of treated TTCF. A phosphate buffered saline (PBS) only injection group was also included as a further negative control. Mice were bled weekly by tail venesection; a booster dose (5 µg TTCF) was given at day 28 and a terminal bleed collected at day 42. Collected serum was analysed for immune response using enzyme linked immunosorbent assay (ELISA).

### ELISA analysis of serum samples

Purified recombinant TTCF was coated at 10 µg/ml, 100 µl/well onto high-binding 96-wells ELISA microtiter plates (Greiner, UK) in Na_2_CO_3_ buffer, pH 9.6 overnight at 4 °C. Plates were washed three times with phosphate buffered saline (PBS) and then blocked for 1 hour in PBS-Tween 0.05% (PBS-T) containing 1% Casein. Plates were washed 3x with PBS-T. Anti-TTCF mouse monoclonal antibody (clone 10G5^20^) at a concentration of 1 µg/ml was added to act as a positive control and allow normalisation across ELISA plates. Fifty times pre-diluted samples (in PBS-T) and 10G5 were serially two-fold diluted before transfer to the ELISA plates and incubated for 1 hour. Plates were then washed 3 times. Peroxidase conjugated goat-anti-mouse IgG (Sigma UK), at 1:10.000 dilution in PBS-T was then added for 1 hour. Plates were washed 4x and freshly prepared tetra-methyl-benzidine (TMB) substrate solution (0.1 M Sodium Acetate pH 6.0, 10 µg/ml TMB, 0.015% H_2_O_2_) added, followed by 10% H_2_SO_4_ to stop the reaction. Absorbances were read at 450 nm and corrected by subtraction of the reference read at 650 nm and normalised to the positive control (10G5 monoclonal antibody) absorption. Statistical analysis was performed using SPSS v13 (IBM, USA), one-way ANOVA with post-hoc Tukey HSD test.

### Time-resolved (ultra) SAXS (ID02, ESRF)

Small Angle X-Ray Scattering (SAXS)^21^ measurements were performed on the Time-Resolved Ultra Small-Angle Scattering beamline ID02 at the ESRF, Grenoble, France^22^. The incident X-ray energy was 12.46 keV and two sample-detector distances were employed: 1.5 m (SAXS) and 10 m (USAXS). SAXS data were acquired using the Rayonix MX-170HS detector with exposure times between 0.01 and 0.03 seconds at room temperature (20 °C).

Pre-hydrolysed TEOS was added to 10 ml of 1 mg/ml TTCF solution at 1:50 (v/v) ratio at pH 7 *ex situ*, initiating the ensilication reaction. Using a sterile syringe, 1 ml of this ensilication mixture was injected into a quartz capillary (Fig. S2) after which the beamline hutch was checked for safety and closed for the start of measurement. We measured the delay on hutch closure to be approximately 1 minute. As a result, the total time delay between the start of the reaction and the first measurements was between 1 and 2 minutes.

The measured 2D patterns, after normalisation by incident flux, sample transmission, and solid angle, were azimuthally averaged to obtain the 1D static scattering profiles as a function of the magnitude of the scattering vector *q* = 4π/λ sin(θ/2), with λ the incident X-ray wavelength (=0.996 Å^−1^) and θ the scattering angle. This gave two overlapping *q*-ranges of 0.0008 ≤ *q* ≤ 0.008 and 0.006 ≤ *q* ≤ 0.5 Å^−1^. The scattering background in each case was measured using Tris buffer and the normalised background subtracted data are represented by *I*(*q*).

### Fitting of SAXS data

Data fitting was done using several models within SASview to probe the various changes observed in the scattering signal. Good residual fits were found using a combination of power law, ellipsoid, broad peak and mass fractal models at different stages of the ensilication process. The ellipsoid model^23^ provided shape information (the polar and equatorial radii, R_polar_ and R_equatorial_ respectively) on the protein and the initial growth of its silica coating. The broad peak model gave a characteristic length scale for scattering consistent with the particle sizes from the ellipsoid fits, and was utilised as a transition model towards the mass fractal growth^24,25^. The latter provided the fractal radius of silica particulates and fractal dimension, R_frac_ and D_f_ respectively, which are indicative of reaction type^26^. All models were assessed on χ^2^ as a goodness-of-fit indicator. Detailed information about the fitting parameters are presented in the Supplementary Information.

### in situ time-resolved SAXS (i22, Diamond Light Source)

Small Angle X-Ray Scattering (SAXS) measurements during ensilication *in situ* were performed on the Time-Resolved Small-Angle Scattering beamline i22 at Diamond Light Source, Didcot, UK.

TTCF at 1 mg/ml in (50 mM Tris pH 7.0) buffer, 25 ml volume, was circulated using a peristaltic pump at 2 ml/min through Teflon tubing with an internal diameter of 1.6 mm. The sample solution passed through a 1.5 mm capillary flow cell in a loop before addition of hydrolysed TEOS via a syringe injector. Both pump and injector were remotely controlled (Fig. S3).

SAXS frames were taken at 1 frame/sec for 120 seconds. Pre-hydrolysed TEOS was added to the sample at 1:50 (v/v) ratio after 3 seconds from start of measurement, so that data on the native protein could be acquired before ensilication began. The incident X-ray energy was 12.4 keV. SAXS data were acquired using the Pilatus P3-2M detector at 2.2 m sample distance with 0.8 seconds exposure time. The collected 2D data were processed using a pipeline setup in the Data Analysis WorkbeNch (DAWN) software^27^. The pipeline was set up with detector calibration, SAXS mask, Poisson error, time with flux and transmission correction. Azimuthal integration produced 1D data in *I* vs *q*, where *q* = 4π/λ sin(θ/2), with λ the incident X-ray wavelength (0.9998 Å) and θ the scattering angle. The *q* range was between 0.008 ≤ *q* ≤ 0.75 Å^−1^.

The experiment was performed at room temperature (20 °C). Background subtraction scattering was done using a double subtraction method (Fig. S4 & Table S1). Empty capillary scattering was subtracted from Tris buffer scattering. The sample SAXS was then processed by subtracting Tris buffer (minus capillary scattering) and capillary scattering only (Table S1).

### Native TTCF SAXS (i22 Diamond and ID02 ESRF)

Native TTCF SAXS at 1 mg/ml in 50 mM Tris buffer pH 7 was measured at both beamlines i22 and ID02 as described for the time-resolved setups. At ID02, native TTCF SAXS was collected by taking 10 frames of 0.03 sec exposure time on a sample consisting of the protein in buffer solution without the addition of silica. These data were processed as described for ESRF data with *q* range 0.006 ≤ q ≤ 0.5 Å^−1^. At i22, native TTCF SAXS was collected during the first 3 seconds of measurement (1 frame/sec) of the *in situ* experiment, before the addition of silica began. These 3 frames were averaged and processed using the aforementioned DAWN pipeline with *q* range 0.008 ≤ q ≤ 0.75 Å^−1^.

## Results and Discussion

In our previous paper we observed the retention of protein primary and tertiary structure via ensilication, including heat treated material, using biochemical methods^14^. In the present study, we are looking at two opposite experimental resolutions with support of evidence found at the protein secondary structure level.

At the molecular level, time-resolved (*in situ*) SAXS and fitting of mathematical models were employed to elucidate protein-silica particle formation^21,28^ and characterise the growth process of the silica tailored coating of TTCF. This is a powerful tool that can assess morphology of proteins and particles formed at the nanometre scale over time.

At the other end of the spectrum, *in vivo* immunisation experiments confirmed feasibility of the end stage application for our methodology, creating an opportunity to solve real-world challenges.

**Figure 1:**
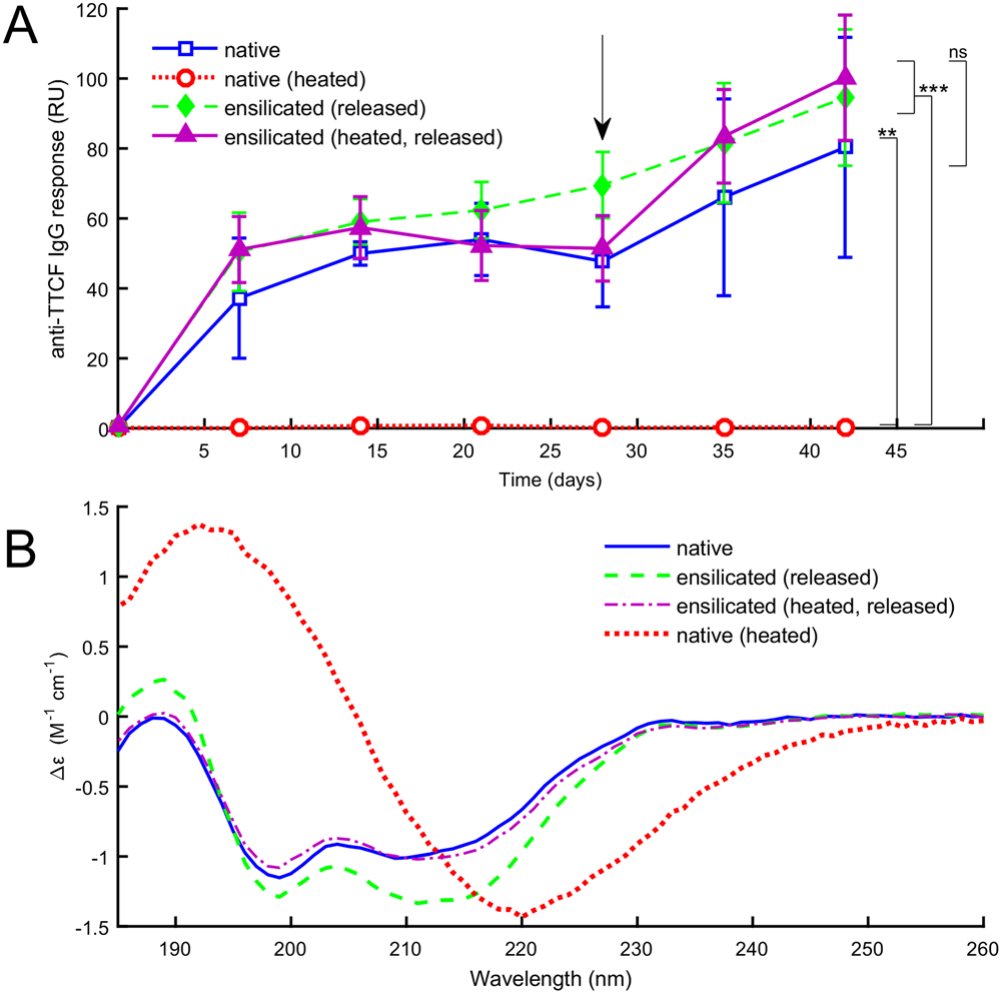
(**A**) Serum IgG response from mice immunized with TTCF after ensilication. Normalized absorbance values against monoclonal 10G5 (RU: relative units). Sample groups consisted of 5 mice which were immunized with 5 µg of TTCF at the start of experiment and boosted at 28 days (arrow). Data are expressed as mean +/− SD (n = 5). Statistical analysis was performed applying one-way ANOVA with post-hoc Tukey HSD test. Comparison vs. native TCCF (ns = nonsignificant) or vs. native TTCF heated to 80 °C to denature and inactivate ((* p ≤ 0.05, ** 0.01 and *** 0.001). (**B**) Circular dichroism (CD) data for native TTCF, for TTCF released from ensilication with and without heat treatment, and for TTCF heated without ensilication. The ensilicated protein retains the same secondary structure features as the native TTCF, while the unprotected protein denatures.

To confirm the protective capacity of ensilication *in vivo*, we immunised mice with native or denatured TTCF, or with TTCF after ensilication, and obtained serum samples. The ensilicated material used during this study was stored in powdered form for 1 month at room temperature and then transported using commercially available means without any specialised equipment. Enzyme Linked Immuno-Sorbent Assay^29^ (ELISA) using native TTCF coated plates were employed to analyse the resultant immune response. As expected from previous studies^30^, we observed an early stage immune response in the mice injected with native TTCF. This immune response was matched when the mice were injected with TTCF which had been stored as ensilicated powder and released prior to immunisation (Fig. 1A). Additionally, flow cytometry analysis of day 42 (post-immunisation) splenocytes (Fig. S5) indicated that ensilication of TTCF had no influence on the measured immune response. CD data confirmed that ensilicated-and-released TTCF preserved the secondary structure characteristics of native TTCF (Fig. 1B). In contrast, TTCF heated without ensilication was denatured and visibly precipitated out of solution after 2 hours at 80 °C. We verified the denaturation of TTCF before precipitation by tracking the CD spectrum during heating (Fig. S6), which showed a dramatic transition from the native state to a denatured state. The CD spectrum of the denatured state is also shown for comparison in (Fig. 1B). Heated TTCF was found not to provoke a specific immune response when injected into mice (Fig. 1A).

The results confirmed the protective capacity of ensilication to maintain normal protein/epitope conformation at ambient temperature, as well as when the ensilicated material was heated at 80 °C for 2 hours. This demonstrates the thermal resilience, ease of use and efficacy of ensilication as a method for biopharmaceutical storage and transport without refrigeration.

Initial assessment of the protein scattering data confirmed a monomeric solution of native TTCF (Fig. S7, S8 & Table S2) with some aggregates also present. These data were fitted using  a power law, to model the upturn at low-*q* representing the aggregate structure, combined with an ellipsoid model for TTCF. Calculated parameters derived from modelling suggest TTCF is a prolate ellipsoid, consistent with the known crystal structure^31^ (Fig. S9 & Table S3). The two independent SAXS experiments performed were intended to provide resolution of the onset and longer evolution of TTCF ensilication.

**Figure 2:**
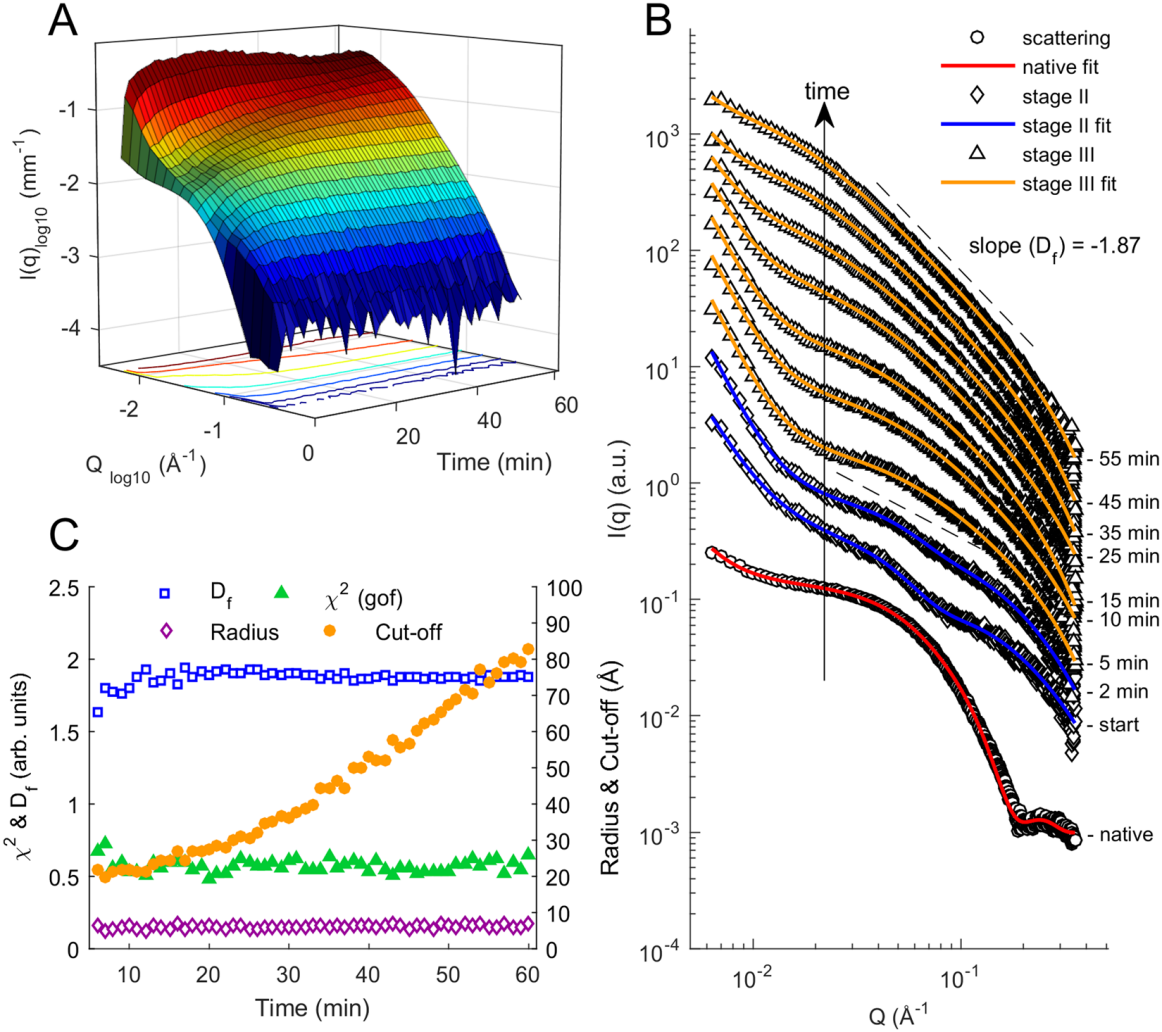
SAXS scattering of TTCF ensilication over time. (**A**) 3D plot of time, scattering vector magnitude *Q*, and intensity *I(Q)* over 60 minutes during the ensilication of TTCF. (**B**) Stacked plot of scattering for selected time points during the experiment. Scattering scaled and truncated at high *q* for clarity (see Fig. S11 for full q-range). Red, blue and orange fittings use a power law + ellipsoid, broad peak + mass fractal, power law + mass fractal model respectively. (**C**) Batch analysis data on mass fractal fitting of acquired SAXS signal. D_f_ = fractal dimension, r (Å) = fractal radius, cut-off length (Å) = static length of aggregating silica particles, χ^2^ = residuals of fit.

Time resolved SAXS (ESRF, ID02) of non-agitated capillary setup TTCF ensilication was used to monitor the ensilication process after *ex situ* initiation, with data collection beginning 1–2 minutes after the initiation of the ensilication reaction and continuing for sixty minutes. After measurement was started, an amorphous peak in the *q*-range of 0.03 ≤ *q* ≤ 0.08 (Å^−1^) was identifiable (Fig. 2) along with a rapid increase in the high-*q* signal. Data from the first few minutes of measurement are well fitted by a broad peak + mass fractal model. The mass fractal represents the growth of the silica aggregate. The broad peak, with a characteristic length scale of around 200 Å between scattering inhomogeneities, represents scattering from protein/silica particles. The broad peak is discussed further in regard to the *in situ*-initiated experiments described below. Over several minutes the fit worsens, with a reduction in the broad peak scattering and an increase in the mass fractal scattering from high to mid-*q*. A model consisting of the combination of power law and mass fractal, with a steadily increasing particle size cut-off, is a better fit to the data from around 5 minutes onwards, consistent with the formation of mass aggregates.

Our interpretation is that individual TTCF-silica nanoparticles form and grow over the first few minutes of the process, and then begin to aggregate into larger clusters. The fractal dimension *D*_f_ of the mass fractal in the fitted models increases over the first few minutes of the experiment from around 1.6 to a steady value of around 1.87 ± 0.05, which is consistent with a diffusion-limited cluster aggregation process^24–26^.

**Figure 3:**
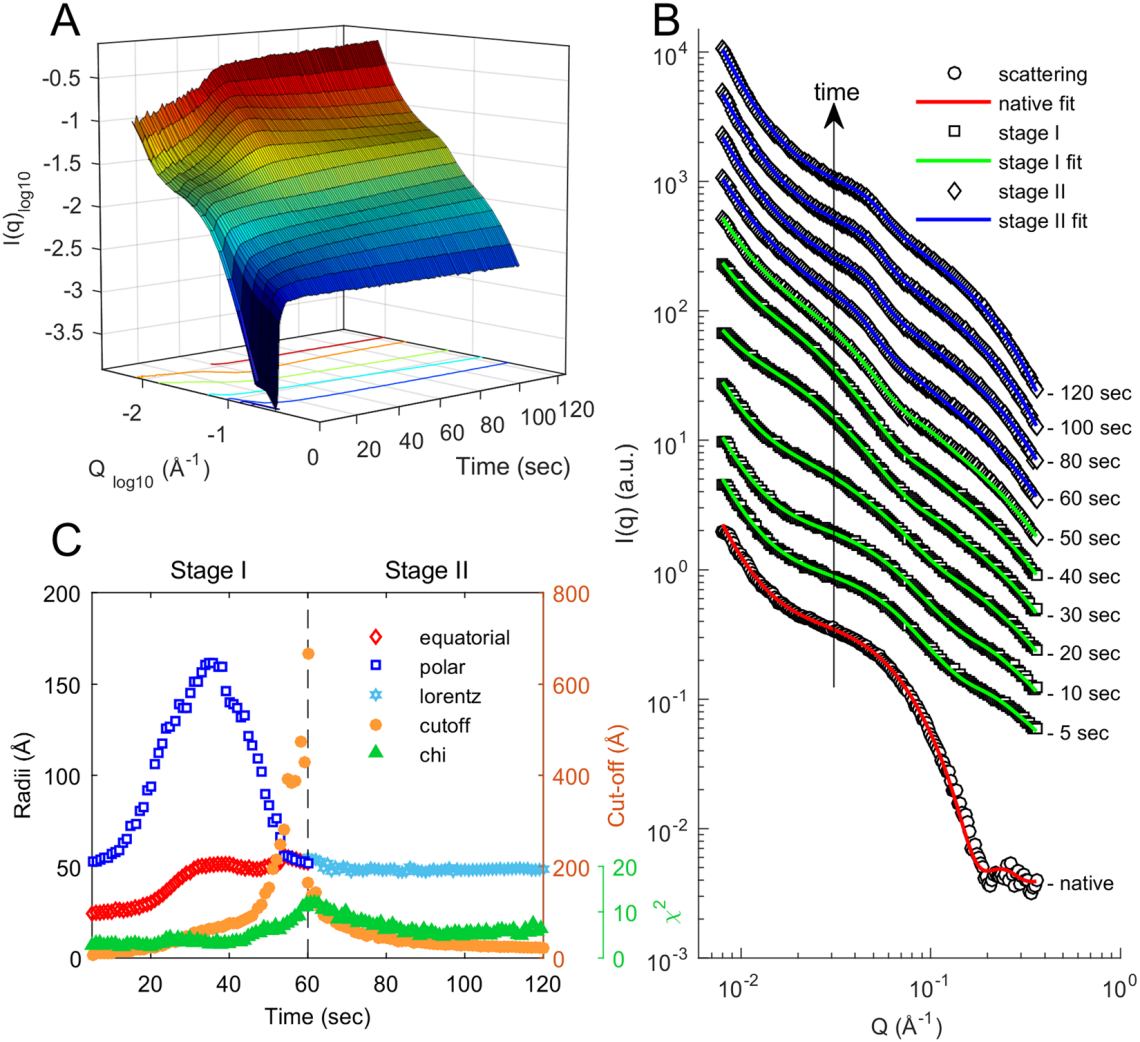
SAXS scattering of continuous flow TTCF ensilication. (**A**) 3D plot of time, scattering vector magnitude *Q*, and intensity *I(Q)* over 120 seconds during the *in situ* ensilication of TTCF. (**B**) Stacked plot of truncated scattering for multiple time points during the experiment (see Fig. S11 for full q-range). Scattering scaled 1 log for each chosen time point. Red, green and blue fittings use a power law + ellipsoid, power law + ellipsoid + mass fractal and broad peak + mass fractal model respectively (**C**) Batch analysis data fit results of acquired SAXS signal. Equatorial: equatorial radius (Å); Polar: polar radius (Å); Lorentz: Lorentz-length, measurable length in an amorphous structure (Å); cut-off length = static length of silica particles coating TTCF (Å), χ^2^ = residuals of fit for stage I & II fit model (Supplementary Information).

In order to monitor the very onset of TTCF ensilication, we also used a flow-cell setup with sample agitation (Diamond Light Source, i22) allowing continuous *in situ* measurement. The *q*-range of 0.008 ≤ *q* ≤ 0.35 Å^−1^ (Fig. 3A) displayed the rapid development of protein-silica particles after addition of pre-hydrolysed TEOS. There is a change in scattering at high *q* (0.20 ≤ *q* ≤ 0.35 Å^−1^, Fig. 3B) and fluctuations in the low-*q* range (0.008 ≤ *q* ≤ 0.04 Å^−1^). The high *q* signal appears immediately after the pre-hydrolysed TEOS is added and indicates silica condensation induced by the presence of charged residues at the surface of the protein^32,33^ (Figs. 3, S8). After approximately 30–40 seconds the protein signal has shifted towards the low *q* range, providing evidence of particle growth caused by ensilication. Supporting this observation is the fractal structure formed at high *q*.

Data from the early stages of this experiment are well fitted by a model combining a power law, representing aggregated protein/silica material; an ellipsoid, representing a protein and the silica coating growing around it as a particle; and a mass fractal, representing fractal growth of the silica network on a larger scale. From around 40 seconds onwards the fit of this model gradually worsens, and by around 60 seconds the scattering has a visibly different profile. The profile is now better described by the combination of the mass fractal with a (Lorentzian) broad-peak model, which also includes a power-law component. Broad peak scattering is typical of soft condensed matter with a complex structure having a characteristic length scale between scattering inhomogeneities. The peak of the Lorentzian, *q*_0_, is consistently found at around 0.03 Å^−1^, corresponding to a characteristic length scale of around 50 Å. This is broadly consistent with the typical diameter of the (protein + silica) ellipsoidal radii of the earlier fit.

The broad peak + mass fractal fitting of the *in situ* experiment are consistent with the corresponding fit to the first minutes of the *ex situ* experiment. An overlay of the SAXS data from the two different experiments at the overlap time of 120 seconds after initiation is shown in Fig. S10. Comparative fits of the ellipsoid and broad-peak models for the *in situ* experiment, with χ^2^ goodness-of-fit values as a function of time, are shown in Fig. S11. Similarly, comparative fits of the broad-peak and power law fits for the *ex situ* experiment are shown in Fig. S12.

The behaviour of the fitting parameters during the first minute of ensilication is particularly interesting, requires careful interpretation and merits further discussion. The polar and equatorial radii of the fitted ellipsoid are initially around 50 and 25 Å respectively, and we take it that the ellipsoid represents the scattering from the individual protein in solution. Over the period of 10–40 seconds, the radii increase rapidly and substantially, with the polar radius tripling in value to around 150 Å at 35 seconds, and the equatorial radius doubling to around 50 Å. In this phase the ellipsoid represents the scattering from a protein and its growing silica coat.

From about 30 to 50 seconds, the cut-off radius of the fitted mass fractal begins to increase linearly. This indicates that particles are starting to aggregate rather than remaining separate. Since some of the scattering from silica is now being represented by the mass fractal, the fitted polar radius of the ellipsoid begins to decline from its maximum as swiftly as it rose. By just after 50 seconds, the fitted radii of the ellipsoid have converged at a single value around 50 Å, while the cut-off radius of the mass fractal is increasing more rapidly. Thus, the period between thirty and fifty seconds after onset appears to mark the transition between a suspension of individual silica-coated proteins and an aggregated cluster thereof. This is followed by the transition to broad-peak scattering.

**Figure 4:**
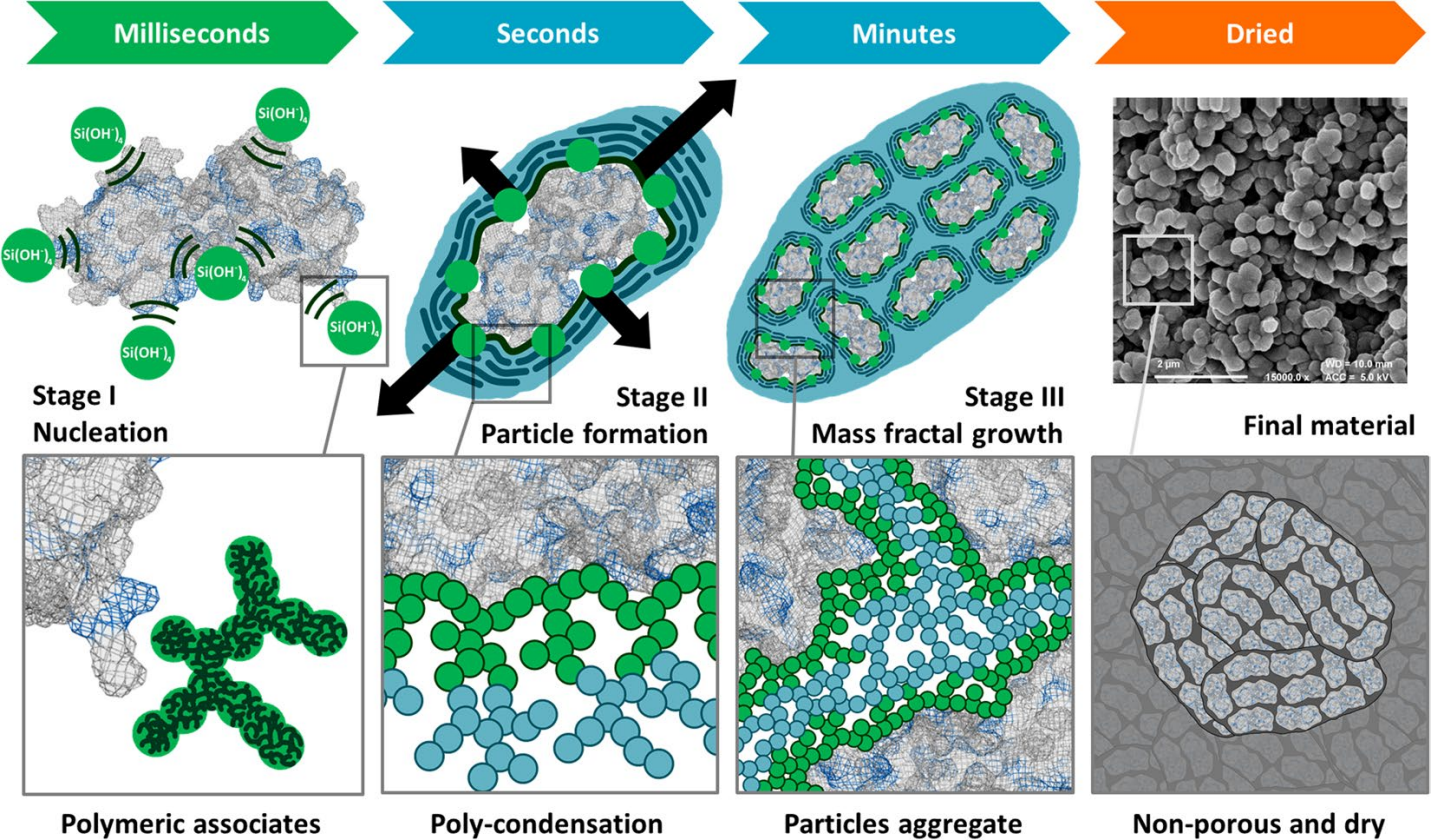
Graphical representation of TTCF ensilication. Ensilication is a continuous process in which silica condenses around proteins, first coating individual proteins and then forming an aggregate. Key stages in the course of this process are illustrated here. The addition of hydrolyzed TEOS (SiOH_4_) to protein in buffer at neutral pH results in nucleation of silica. This is induced via electrostatics which initiates ensilication at positively charged external residues (*e.g*. lysine, arginine, in blue) present on the protein that is attracting the negatively charged silica (stage I). The subsequent condensation and polymerization of polymeric silica species results in the silica coating of individual proteins (stage II). The resulting charge imbalance then leads to stabilization via aggregation and mass fractal growth (stage III). Vacuum filtration of the then turbid solution results in dried powder material containing aggregates of protein loaded silica nanoparticles (FE-SEM at 15,000x magnification).

Based on the data and fittings reported in Figs. 2 and 3, we can infer a consistent physical picture of the ensilication process, illustrated schematically in Fig. 4:

Stage I: Initial nucleation of silica around charged sites on the protein surface; rapid (within seconds) growth of the silica coating by further polymerisation.

Stage II: On a timescale of tens of seconds: silica condensation continues to completely coat the individual proteins, leading to aggregation.

Stage III: On a timescale of minutes: aggregation of nanoparticles and further condensation of silica in a diffusion limited cluster aggregation (DLCA) type process^34^. A fractal cluster made up of many individual nanoparticles makes up the final product, as confirmed by FE-SEM imaging of the dried powder after completion of the ensilication process (shown in Fig. 4). We emphasise that ensilication is a continuous process in which silica condenses around proteins, first coating individual proteins and then forming an aggregate. The stages described here are not discrete but evolve continuously from one to the next.

Our findings are further supported by SAXS carried out a lower *q*-range (0.0008 to 0.05 Å^−1^) displaying large particle formation after silica addition (Figs. S13, S14). Particle sizes increasing to a stable range of 2000–2200 Å (200–220 nm) are observed, consistent with the ~200 nm particle sizes observed previously in the ensilication of a different protein, lysozyme^14^. These data suggest that once TTCF is coated with silica, the TTCF-silica particles are prone to aggregate and form a stable complex. FE-SEM imaging of the final dried material confirms these sizes (Fig. 4). Overall, the SAXS data provides evidence of a fast DLCA process where, once the protein is coated, on average 2000 Å (200 nm) aggregates of protein-silica particles will form if the ensilication is allowed to proceed. The preparation of smaller, less aggregated particles would be possible by interruption of the ensilication process within the first few minutes after onset.

While ensilication is a suitable method for protein preservation, it currently requires laboratory conditions for the silica removal and subsequent dialysis, which would not be suitable in real-world clinical conditions, particularly those in low-income countries. Therefore, for injectable biopharmaceuticals, such as vaccines, we are working towards alternative release methods included in an all-in-one device which we hope to prototype in the near future.

### Ethics Statement

Mice were housed at the comparative biology centre, Newcastle University. All experiments were conducted in accordance with institutional guidelines, approved by Animal Welfare Ethical Review Body (AWERB) and by the Home Office of the United Kingdom, under the auspices of project license P35D9C60C.

## Conclusions

We believe that this new methodology has given rise to another solution for biopharmaceutical stabilisation. In this study, we have studied ensilication using SAXS and demonstrated the protective capacity of ensilication via *in vivo* analysis, i.e. the immunogenicity of intact TTCF is maintained. All biopharmaceuticals have unique functions that require different environments for their operations. Therefore, for some, lyophilisation might be the most appropriate means of stabilising and transporting the bioactive compounds. We propose that those which are not compatible with lyophilisation could be remediated via ensilication.

## Supplementary Information

Supplementary Information.
