# Supplementary material for: Ensilicated tetanus antigen retains immunogenicity: in vivo study and time-resolved SAXS characterization
Source: Sci Rep. 2020 Jun 8;10:9243. doi: 10.1038/s41598-020-65876-3 (PMC7280242; doi:10.1038/s41598-020-65876-3)
Supplement: Supplementary file 1 — Supplementary Information. [file 41598_2020_65876_MOESM1_ESM.docx]

**SUPPLEMENTARY INFORMATION FOR:**

**Ensilicated tetanus antigen retains immunogenicity: *in vivo* study and time-resolved SAXS characterisation**

A. Doekhie ^a^, R. Dattani ^b^, Y-C. Chen ^a^, Y. Yang ^c^, A. Smith ^d^, A. P. Silve ^a^, F. Koumanov ^e^, S.A. Wells ^g^, K.J. Edler ^a^, K.J. Marchbank *^c^, J.M.H. van den Elsen *^f^ and A. Sartbaeva *^a^

*^a^ Department of Chemistry, ^b^ European Synchrotron Research Facility, ^c^ Institute of Cellular Medicine, ^d^ Diamond Light Source, ^e^ Department for Health, ^f^ Department of Biology and Biochemistry, ^g^ Department of Chemical Engineering.*

*^a, e, f, g^ University of Bath, Claverton Down, Bath, BA2 7AY, United Kingdom.*

*^b^ ESRF, 71* *avenue des Martyrs, CS 40220, 38043 Grenoble Cedex 9, France.*

*^c^ Newcastle University, Medical School, Newcastle upon Tyne, NE2 4HH, United Kingdom.*

*^d^ Diamond Light Source Ltd, Harwell Campus, Didcot, OX11 0DE, United Kingdom.*

* *share senior authorship*

All correspondence and requests should be addressed to A.S. ([a.sartbaeva@bath.ac.uk](mailto:a.sartbaeva@bath.ac.uk)).

Contents

[PURITY OF NATIVE TTCF FOR SAXS 3](#_Toc22555462)

[Figure S1. SDS-PAGE of purified rTTCF batches #1 - 4 for SAXS analysis. 3](#_Toc22555463)

[Figure S2. Capillary setup for SAXS experiment at ESRF beamline ID02. 4](#_Toc22555464)

[Figure S3. Flow cell setup for in situ SAXS experiment at Diamond Light Source beamline i22. 4](#_Toc22555465)

[Table S1. Detailed overview of sample runs during the SAXS beamtimes at both beamlines. 4](#_Toc22555466)

[UNSUBTRACTED SAXS SCATTERING 5](#_Toc22555467)

[Figure S4. Unsubtracted SAXS scattering profiles for protein and backgrounds. 5](#_Toc22555468)

[FLOW CYTOMETRY OF *IN VIVO* DAY 42 POST-IMMUNIZATION 6](#_Toc22555469)

[Figure S5. Flow cytometry analysis of Day 42 post immunisation splenocytes. 7](#_Toc22555470)

[NATIVE TTCF DENATURATION MEASURED BY CIRCULAR DICHROISM 8](#_Toc22555471)

[Figure S6. CD spectra of native TTCF subjected to a 5 °C interval temperature ramp from 5 – 85 °C. 8](#_Toc22555472)

[SAXS ANALYSIS 9](#_Toc22555473)

[P(r), Guinier and molecular weight analysis 9](#_Toc22555474)

[Figure S7. SAXS native TTCF protein analysis. 10](#_Toc22555475)

[Figure S8. Direct fitting of native TTCF experimental SAXS data 11](#_Toc22555476)

[Table S2. Molecular weight analysis for TTCF scattering. 12](#_Toc22555477)

[NATIVE TTCF FIT 13](#_Toc22555478)

[Figure S9 & table S3. TTCF fitting using an ellipsoidal model. 13](#_Toc22555479)

[Figure S10. SAXS scattering overlap at 120 seconds 14](#_Toc22555480)

[TTCF ENSILICATION FITS 15](#_Toc22555481)

[STAGE I 15](#_Toc22555482)

[STAGE II 16](#_Toc22555483)

[Figure S11. Comparative fits of the Stage I and Stage II models to data from the first two minutes of *in-situ*-initiated ensilication. 17](#_Toc22555484)

[STAGE III 18](#_Toc22555485)

[Figure S12. Comparative fits of the Stage II and Stage III models to data from *ex-situ*-initiated ensilication. 18](#_Toc22555486)

[FIT TABLES – PARAMETER OUTPUT 20](#_Toc22555487)

[TTCF ENSILICATION SUPPLEMENTARY PLOTS 23](#_Toc22555488)

[Figure S13. Full q-range of TTCF ensilication for Diamond and ESRF. 23](#_Toc22555489)

[Figure S14. Guinier-Porod analysis of 0.0008<q<0.08 A-1 range SAXS 1D data. 24](#_Toc22555490)

# PURITY OF NATIVE TTCF FOR SAXS

FASTA sequence recombinant TTCF^1^ used for MODELLER^2,3^ with 1a8d^4^ TTCF crystal structure

| 10 | 20 | 30 | 40 | 50 |
| --- | --- | --- | --- | --- |
| MGHHHHHHHH | HHSSGHIEGR | HMLDNEEDID | VILKKSTILN | LDINNDIISD |
| 60 | 70 | 80 | 90 | 100 |
| ISGFNSSVIT | YPDAQLVPGI | NGKAIHLVNN | ESSEVIVHKA | MDIEYNDMFN |
| 110 | 120 | 130 | 140 | 150 |
| NFTVSFWLRV | PKVSASHLEQ | YGTNEYSIIS | SMKKHSLSIG | SGWSVSLKGN |
| 160 | 170 | 180 | 190 | 200 |
| NLIWTLKDSA | GEVRQITFRD | LPDKFNAYLA | NKWVFITITN | DRLSSANLYI |
| 210 | 220 | 230 | 240 | 250 |
| NGVLMGSAEI | TGLGAIREDN | NITLKLDRCN | NNNQYVSIDK | FRIFCKALNP |
| 260 | 270 | 280 | 290 | 300 |
| KEIEKLYTSY | LSITFLRDFW | GNPLRYDTEY | YLIPVASSSK | DVQLKNITDY |
| 310 | 320 | 330 | 340 | 350 |
| MYLTNAPSYT | NGKLNIYYRR | LYNGLKFIIK | RYTPNNEIDS | FVKSGDFIKL |
| 360 | 370 | 380 | 390 | 400 |
| YVSYNNNEHI | VGYPKDGNAF | NNLDRILRVG | YNAPGIPLYK | KMEAVKLRDL |
| 410 | 420 | 430 | 440 | 450 |
| KTYSVQLKLY | DDKNASLGLV | GTHNGQIGND | PNRDILIASN | WYFNHLKDKI |
| 460 |  |  |  |  |
| LGCDWYFVPT |  |  |  |  |

| 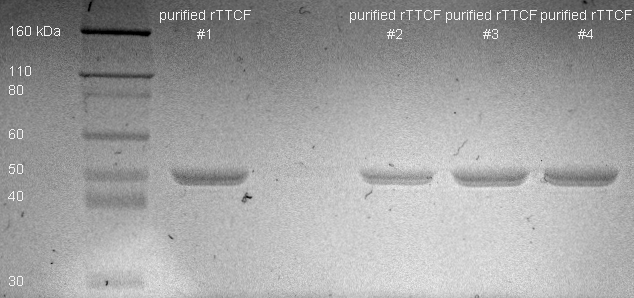 | | | | | | | |  |  |  |  |
| --- | --- | --- | --- | --- | --- | --- | --- | --- | --- | --- | --- |
| **Figure S1. SDS-PAGE of purified rTTCF batches #1 - 4 for SAXS analysis.** Sample lanes loaded with 5 µg of His-Tag purified TTCF normalised to 1 mg/ml in 50 mM Tris buffer pH 7. Concentration determined using commercial BCA protein assay. Molecular weight ladder in kilodalton (kDa) is indicated on the left. | | | | | | | |  |  |  |  |
| **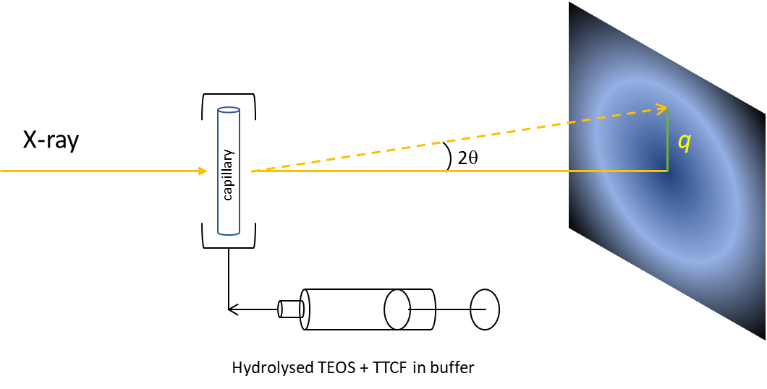TTCF SAXS SAMPLE SETUP** | | | | | | | | | | |  |
| Figure S2. Capillary setup for SAXS experiment at ESRF beamline ID02. Pre-hydrolysed TEOS was added to TTCF in buffer and taken up into a syringe. The sample was then injected into a capillary and the experiment thereafter was started. | | | | | | | | | | |  |
| 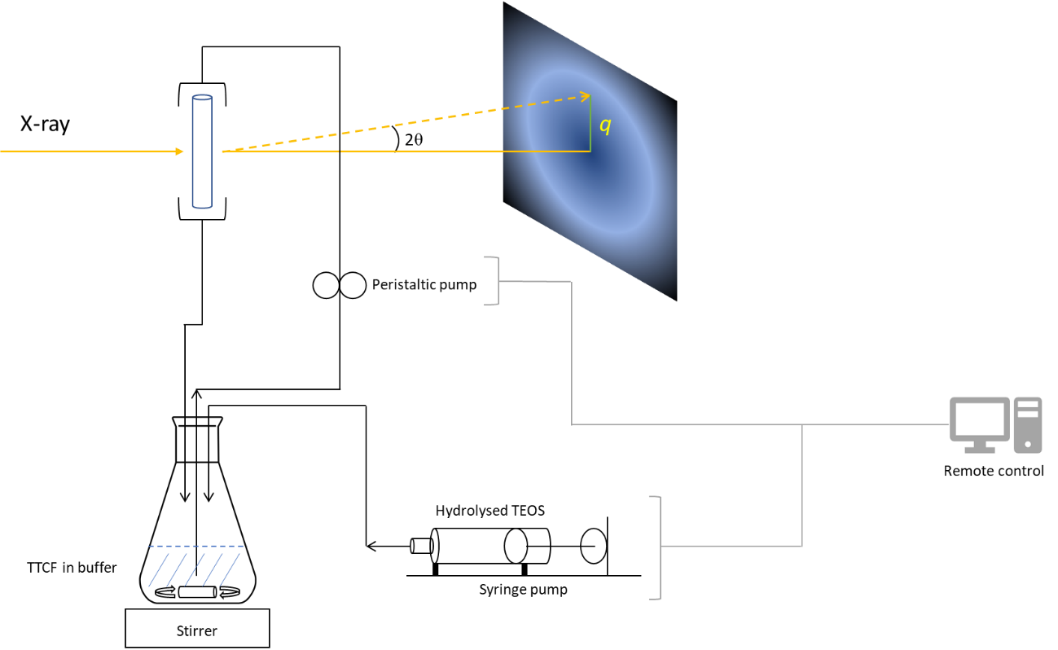 | | | | | | | | | | |  |
| Figure S3. Flow cell setup for in situ SAXS experiment at Diamond Light Source beamline i22. Remote controlled syringe pump injected a specific amount of hydrolysed TEOS into the protein solution which was being agitated using a magnetic stirrer. The peristaltic pump flowed the solution through the flow-cell where frames were taken on the evolution of silica condensation with TTCF involved. | | | | | | | | | |  |  |
| **Sample** | | **Conc.** (mg/ml) | **ratio**  (v/v) | **pH** | **type** | **distance**  m | **q-range**  Å^-1^ | **total time** | | |  |
| ensilication | | 1 | 1:50 | 7 | flow cell (i22) | 2.2 | 0.008 – 0.75 | 900 sec. | | |  |
| ensilication | | 1  1 | 1:50  1:50 | 7  7 | capillary (ID02)  capillary (ID02) | 1.5  10 | 0.006 – 0.51  0.0007 – 0.077 | 60 min  45 min | | |  |
| native TTCF | | 1 | - | 7 | flow cell (i22 | 2.2 | 0.008 – 0.75 | - | | |  |
| native TTCF | | 1 | - | 7 | capillary (ID02) | 1.5 | 0.006 – 0.51 | - | | |  |
| released TTCF | | <1 | - | 7 | capillary (ID02) | 1.5 | 0.006 – 0.51 | - | | |  |
|  | Table S1. Detailed overview of sample runs during the SAXS beamtimes at both beamlines. | | | | | | | | | | |

# UNSUBTRACTED SAXS SCATTERING

| 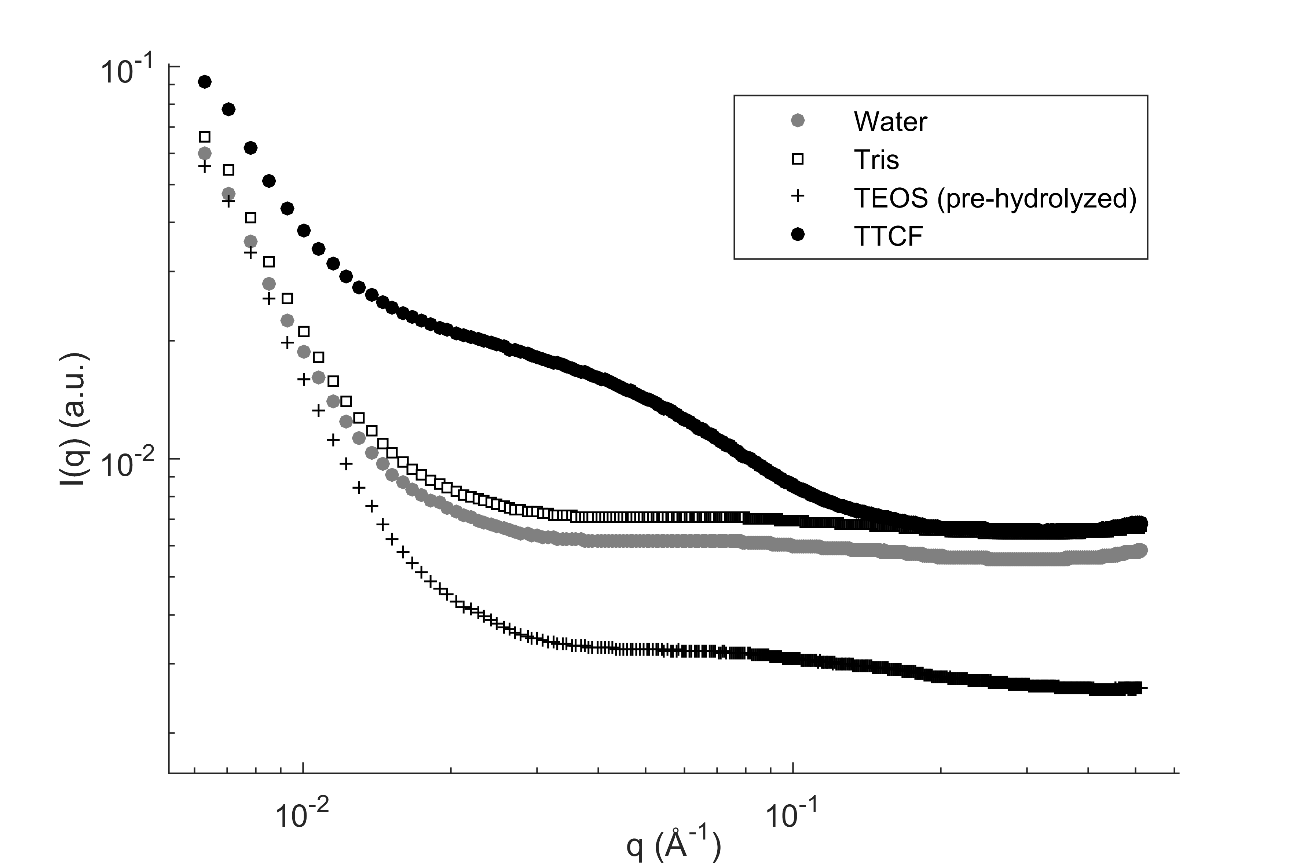 |
| --- |
| Figure S4. Unsubtracted SAXS scattering profiles for protein and backgrounds. |

# FLOW CYTOMETRY OF *IN VIVO* DAY 42 POST-IMMUNIZATION

| 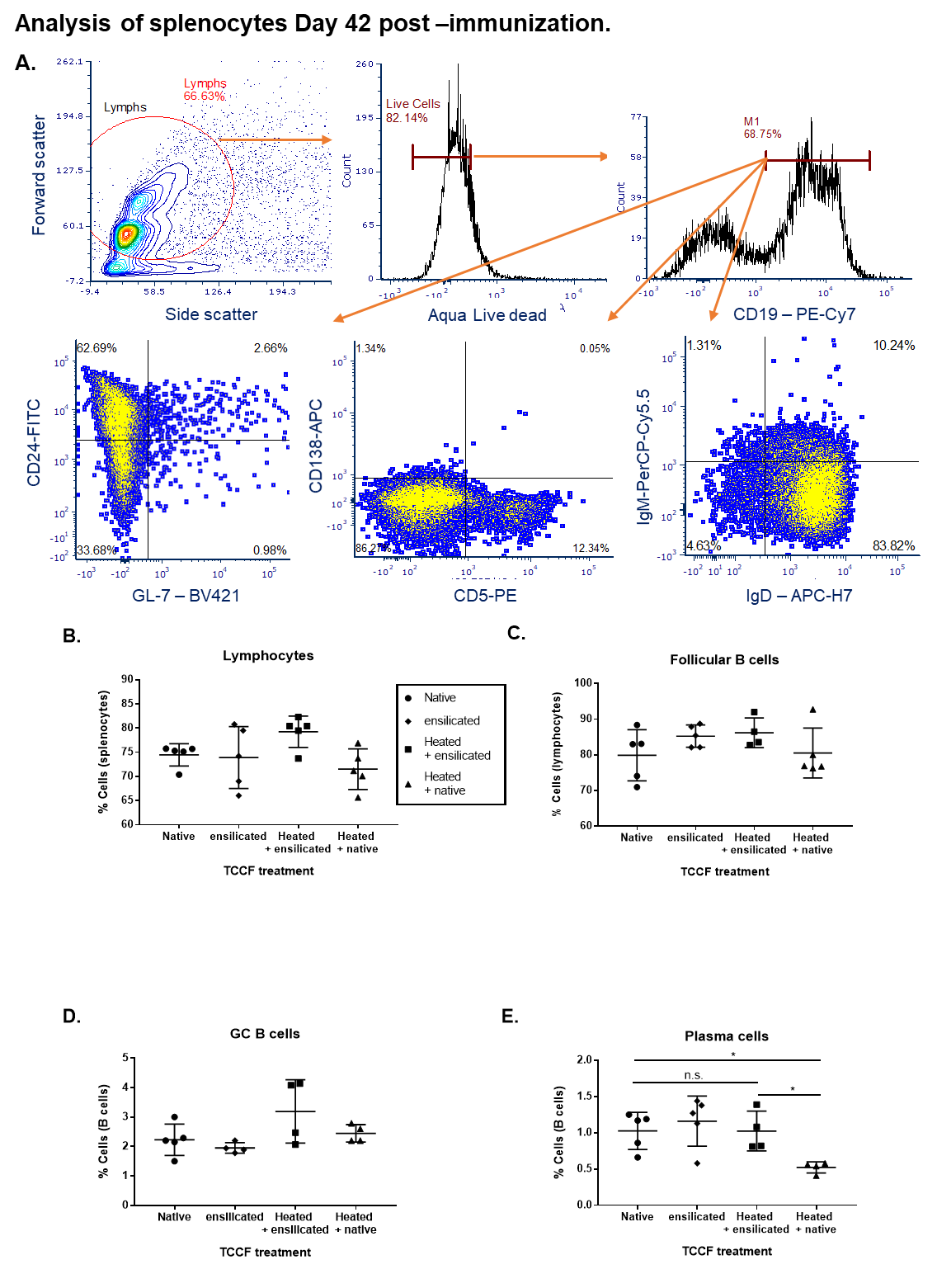 |
| --- |
| Figure S5. Flow cytometry analysis of Day 42 post immunisation splenocytes. All mice in this study are on the C57BL/6 (B6) genetic background and splenocytes were isolated from dissected spleens at Day 42 post initial immunisation (14 days after boost) through crushing between 2 frosted glass slides and resuspension to 15ml in ice cold RPMI. After washing, cells were placed in fetal bovine serum (FBS) containing 10% dimethyl sulfoxide (DMSO) and cooled down to -80 °C for storage. At a later date, tubes were rapidly defrosted under running hot water and the contents placed into 20 mls room temperature RPMI. Tubes were centrifuged at 300 x *g* at 4 °C for 5 min and then suspended in 20 ml ice cold RPMI. After counting, cells were adjusted to 1x10^6^ cells / ml and pelleted. Cells were suspended in PBS/1% BSA/1mM EDTA/0.01% Na Azide (flow buffer) supplemented with BD cell staining buffer, 1 µg/ml of Fc block solution (anti-CD16/32 BD) and Aqua live dead according to manufacturer’s instructions (BD & Invitrogen, respectively). After incubation for at least 15 min, 100 µl of cells in this mixture were mixed with 100 µl of antibody mix and incubated on ice in the dark for 30 min. The antibody mix was CD24- FITC, CD5 – PE, Biotin CD138 followed by SA-APC, IgM-PerCP-Cy5.5, CD19-PE^cy7^, IgD-APC-H7 and GL-7 – BV421. Cells were washed three times, 300 x *g* at 4 °C and cells were suspended in flow buffer containing 2% PFA. Compensation beads (BD Biosciences) were used according to manufacturer’s guidance to allow the FACSCanto to be set up and 5000 CD19^+^ events were collected. Analysis of the flow cytometry data was carried out using FCS express 6 (De novo Software) followed by plotting and statistical analysis in GraphPad Prism7. (A). Shows representative plots and flow cytometry analysis tree. (B) Percentage of splenocytes in the lymphocyte gate as determined by forward and side scatter profile. (C) Percentage CD19^+^IgM^lo^IgD^+^ (follicular B) cells in the lymphocyte gate. (D). Percentage of CD19+GL-7+ (activated germinal centre) cells in the lymphocyte gate and (E) Percentage of CD19+CD138+ (plasma) cells in the lymphocyte gate. Overall, this data suggests ensilication of TTCF has no major influence on B cell numbers or immune response per se. Only the plasma cell population was at a significantly lower percentage in response to the denatured (native + heated) TTCF after immunisation when compared to native TTCF, ensilicated and released TCCF (ensilicated) or ensilicated, heated and then released (ensilicated + heated) TTCF. |

# NATIVE TTCF DENATURATION MEASURED BY CIRCULAR DICHROISM

| 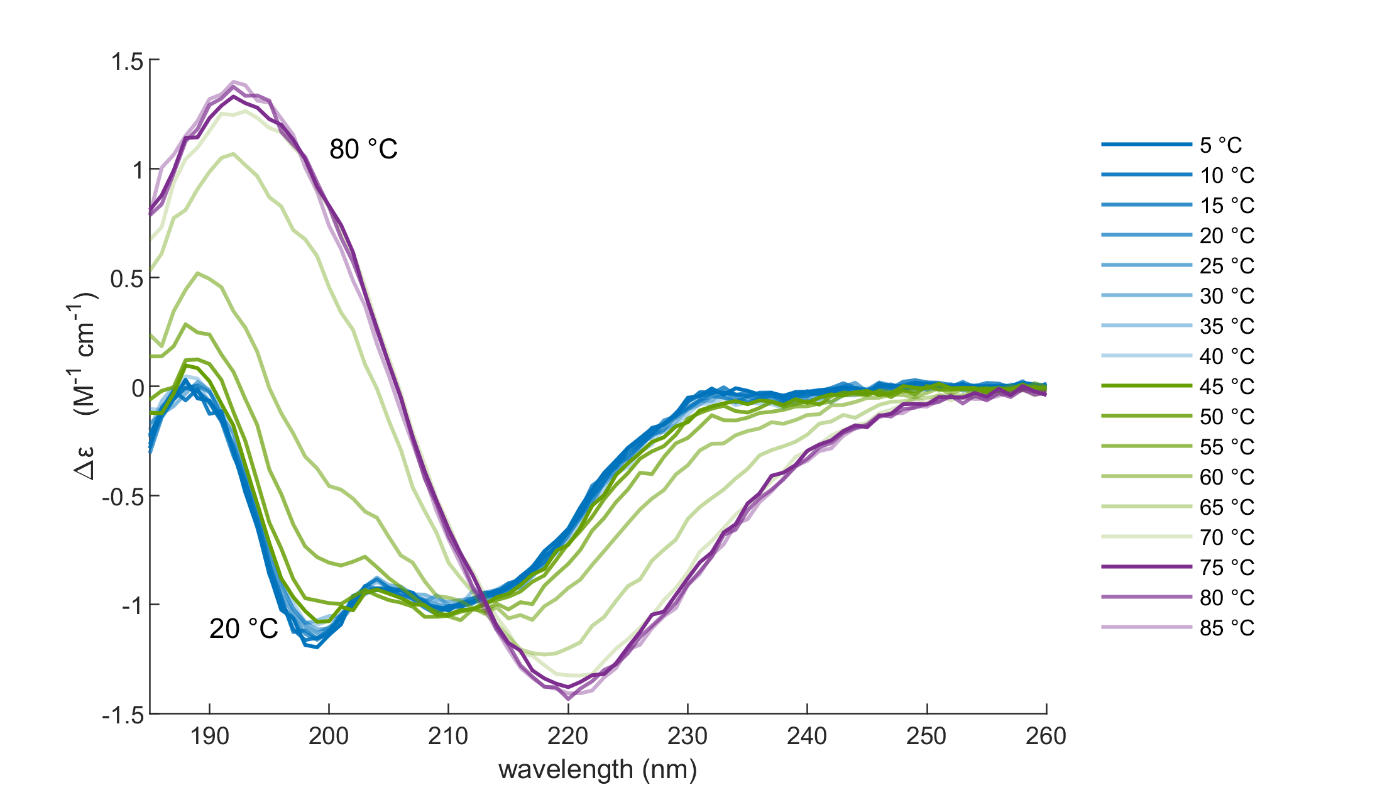 |
| --- |
| Figure S6. CD spectra of native TTCF subjected to a 5 °C interval temperature ramp from 5 – 85 °C. Native TTCF CD was measured between 260 – 185 nm for the duration of the set interval. Each measurement included 2 seconds measurement time per wavelength with a step size of 1 nm and bandwidth of 2 nm. Data were normalised by sample concentration and output in ∆ε (delta epsilon, M^-1^ cm^-1^). The shift in CD spectra observed between 50 and 70 °C depicts the denaturation of TTCF. |

# SAXS ANALYSIS

All mathematical models here will be described up to an appropriate level. For more detailed information we refer to the SASview (www.sasview.org) model documentation with appropriate references stated.

## P(r), Guinier and molecular weight analysis

In order to develop a starting model before carrying out the *in situ* and *ex situ* ensilication SAXS experiments, we assess the morphology of native TTCF in solution. Experimental scattering data on the native protein, obtained at both ESRF and Diamond, are here compared to the simulated SAXS scattering of the crystal model^5,6^. Additionally, in the course of the experiment at Diamond, we obtained data on a sample of TTCF released from ensilication and allowed to stand (for approx. 1 hour) in the release medium, consisting of Tris buffer with fluoride and dissolved silica.

The morphology was obtained by using a real-space inversion P (r)^7^ function and Guinier^8^ approximation of the acquired background-subtracted SAXS signal pattern between 0.006<*q*<0.51 Å^-1^ (ID02) and 0.008<*q*<0.35 Å^-1^ (i22) (fig. S1, A):

$$\left[ Guinier \right] I(q)=\text{scale}\cdot\exp[\frac{-Q^{2}R_{g}^{2}}{3}]+\text{background}$$

| 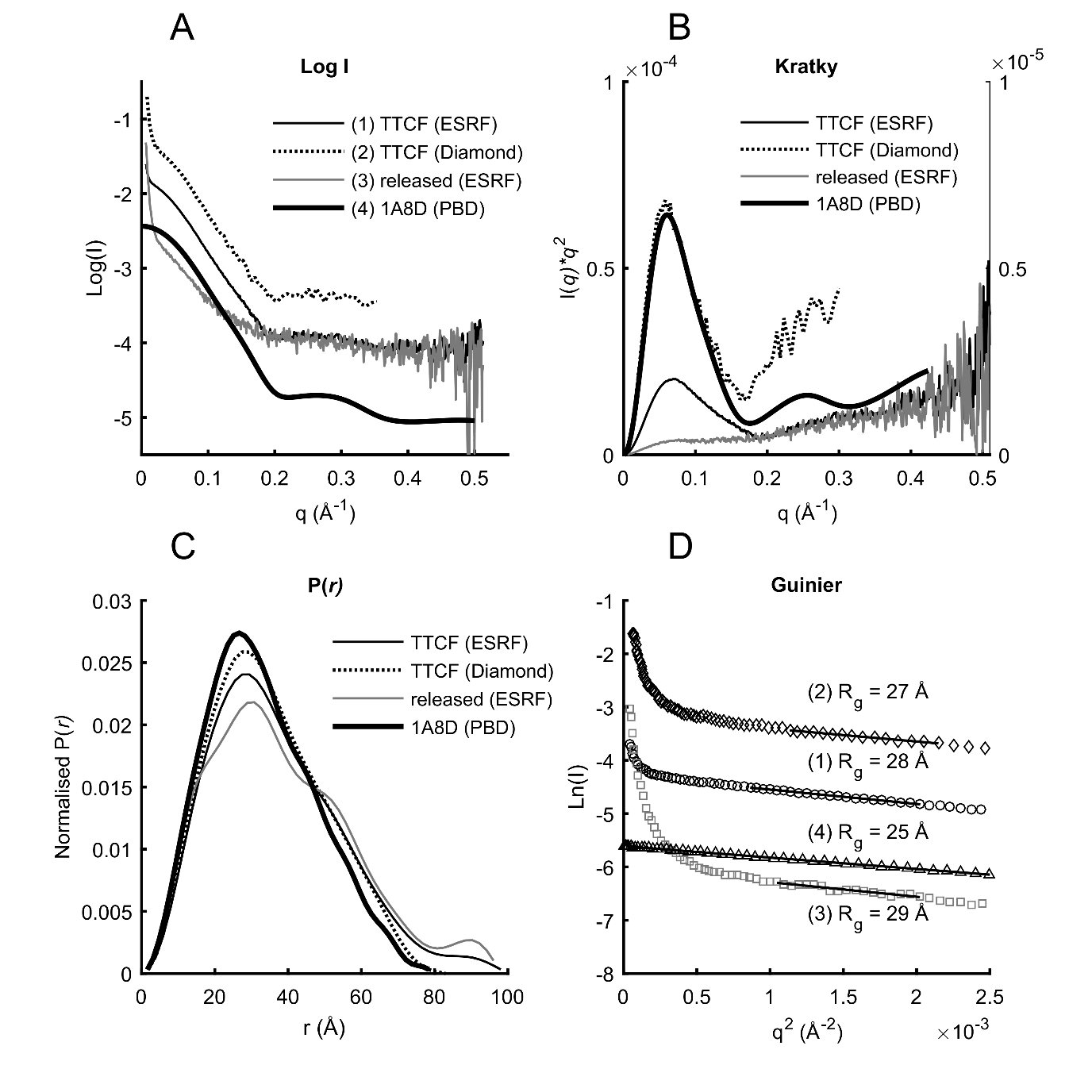 |
| --- |
| Figure S7. SAXS native TTCF protein analysis. (A) Log intensity plot of obtained scattering for 1 mg / ml native solution. Simulated scattering calculated from crystal structure PDB: 1A8D. (B) Kratky plot of native, released and simulated scattering from crystal structure. Plot demonstrates folded protein with flexible region. (C) P(r) plot of previous samples (D) Guinier approximation from scattering between 0.006<q<0.05 Å^-1^ with R_g_ stated for each sample. |

where R_g_ is calculated via slope analysis at small *q*. The R_g_ (fig S1, D) obtained for native TTCF (ID02) is 28 Å, (i22) 27 Å and released TTCF is 29 Å. The observed sizes are in agreement with the homologous (96.7% sequence overlap) published crystal structure of TTCF ^4^ (PDB: 1A8D) with an R_g_ of 25 Å. and confirms the P (r) function derived shape of an ellipsoidal particle (fig. S1, C).

Supporting the above, we directly fitted the 1A8D based model to the experimental SAXS data of native TTCF. Using MODELLER^2,3^, we filled in the missing amino acid constituents of our recombinant TTCF sequence and created a new homologous PDB model. This was run through the FoXs^6^ scattering algorithm that directly fitted our experimental data, shown below. Both data sets showed reasonable agreement with the simulated scattering model. This confirms that 1A8D is an appropriate model to derive structure based parameters from.

| 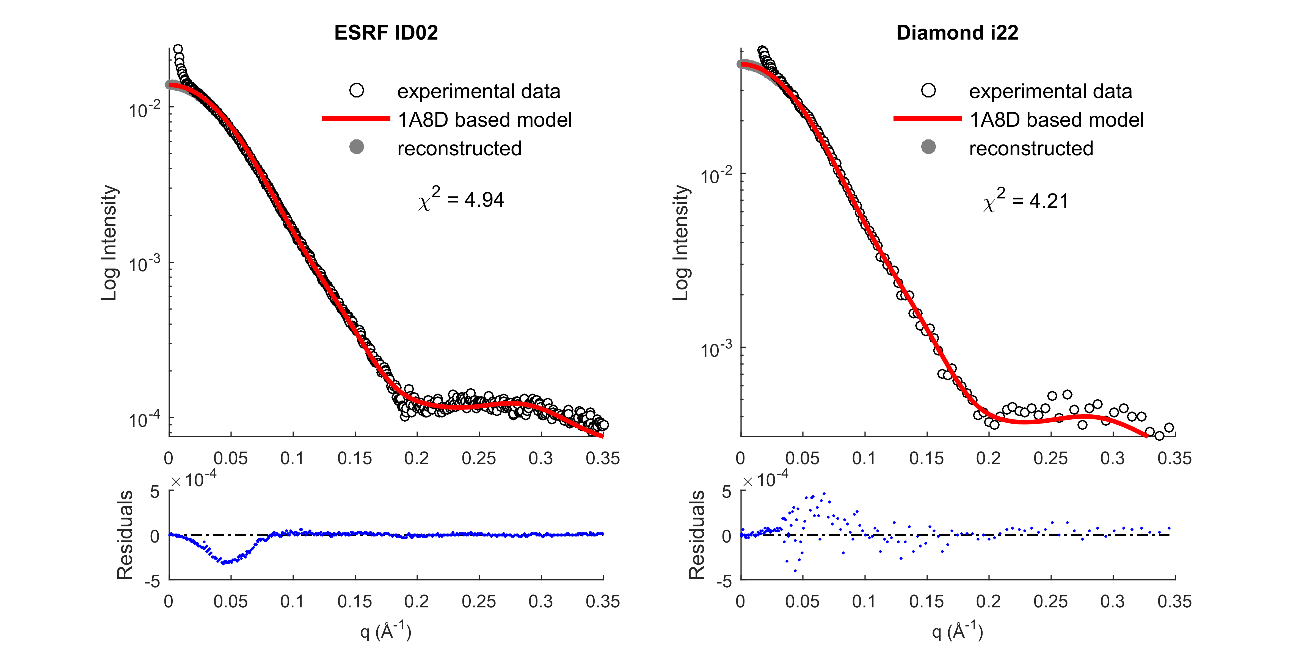 |
| --- |
| Figure S8. Direct fitting of native TTCF experimental SAXS data **with simulated SAXS of 1A8D based model.** The experimental SAXS data was reconstructed at low-q for direct fitting with 1A8D based model. This was created using MODELLER to extend the sequence with our recombinant version. FoXs was utilised to directly fit both data sets to the TTCF crystal model. χ^2^ represents goodness-of-fit. |

Some differences in scattering intensity are observed due to environmental differences (beamline setup) or concentration variations. However, the two experiments on native protein show consistency in the particle size and morphology and indicate a monodisperse solution. The data on the protein released and allowed to stand are noisy due to the low concentration of protein in the released solution and to scattering from silica. The scattering data in this case indicate polydispersity, with protein aggregation and possible unfolding. This indicates the care that must be taken when handling protein that is no longer protected by ensilication. In the *in vivo* experiments discussed in the main MS, the released protein was dialysed out of the release medium; circular dichroism data show the retention of the folded structure, and the immune assay shows that the protein retains its biological activity. This is consistent with our previous study^9^ showing retention of structure and biological function in proteins released from ensilication.

Molecular weight analysis of SAXS scattering was done using the SAXSMoW2 method^10^ . This involved the calculation of the protein volume V that can then be used in the following:

$$MW\left[ kDa \right]=\frac{\rho_{m}\left[ g/{\mathrm{cm}^{3}} \right]V\left[ \mathrm{cm}^{3} \right]}{1.662\cdot{10}^{-21}\left[ g/{kDa} \right]}$$

where $\rho_{m}$= 1.37 g/ml. The method calculated monomeric states for TTCF scattering and agrees with the linearity of the Guinier approximation. The selected upper integration limit used in the molecular weight analysis was defined as: $q_{m}=8/R_{g}$.

| **SAXS MW analysis**^5,6,10^ | **1A8D** | **Native (ESRF)** | **Native (Diamond)** | **Released & let stand** |
| --- | --- | --- | --- | --- |
| Sequence weight (kDa): | 52.22036 | 53.5455 | 53.5455 | 53.5455 |
| Found molecular weight: (kDa) | 45.594 | 48.598 | 41.918 | 39.014 |
| Discrepancy (%): | 14.53 | 10.18 | 27.74 | 37.25 |
| Oligomeric state: | monomer | monomer | monomer | polydisperse |
| Table S2. Molecular weight analysis for TTCF scattering. | | | | |

# NATIVE TTCF FIT

Fitting of native TTCF in solution (i22 & ID02) was done using an ellipsoidal model^11^ (with power law added to compensate for protein aggregates) reveals similar values at both beamlines. Calculations were done using the sum of:

$$I\left( q \right)=\text{scale}\cdot q^{-\text{power }}+ P(q,\alpha)+\text{background}$$

where: $P(q,\alpha)=\frac{\text{scale}}{V}F^{2}\left( q,\alpha\right))$

The ellipsoidal form factor *P(q,*$\alpha$*)* includes the Fourier transform:

$$F(q,\alpha)=\frac{3\Delta\rho V(\sin[qr(R_{p},R_{e},\alpha)]-\cos[qr(R_{p},R_{e},\alpha)])}{[qr(R_{p},R_{e},\alpha)]^{3}}$$

where R_p_: polar radius, R_e_: equatorial radius,$\Delta\rho$: density of particle, α: axis angle, $V$: ellipsoid volume;

$V=(4/3)\pi R_{p}R_{e}^{2}$.

From the analysis it is reported that TTCF is a prolate ellipsoid as its polar radius is larger than its equatorial. The scattering length density (SLD) for TTCF was calculated using the molecular density (1.37 g/ml) and its formula which provided an SLD of 12.4681 x 10^-6^/Å^2^. Both radii have similar sizes and confirm the reproducibility for the expression of TTCF. Scattering intensity does present variations which are due to different beamline setups (fig. S4, S5).

| 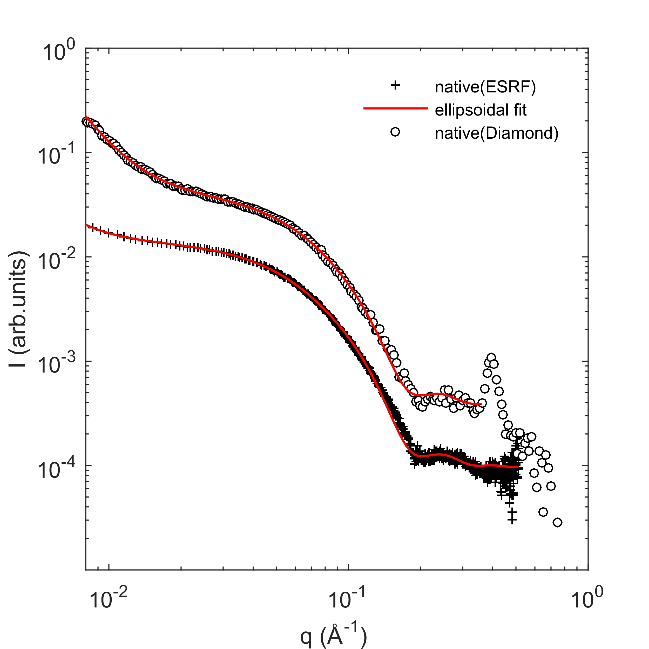 | \| **FITTING** \| **unit** \| **TTCF (ESRF)** \| **TTCF (Diamond)** \| \| --- \| --- \| --- \| --- \| \| **scale** \| N/A \| 13.10 x 10^-5^ \| 42.12 x 10^-5^ \| \| **background** \| cm^-1^ / a.u. \| 9.50 x 10^-5^ \| 3.30 x 10^-5^ \| \| **SLD** (protein) \| x 10^-6^/Å^2^ \| 12.47 \| 12.47 \| \| **SLD** (solvent) \| x 10^-6^/Å^2^ \| 9.44 \| 9.44 \| \| **radius** (polar) \| Å \| 61 \| 58 \| \| **radius** (equatorial) \| Å \| 22 \| 22 \| |
| --- | --- | --- | --- | --- | --- | --- | --- | --- | --- | --- | --- | --- | --- | --- | --- | --- | --- | --- | --- | --- | --- | --- | --- | --- | --- | --- | --- | --- | --- |
| Figure S9 & table S3. TTCF fitting using an ellipsoidal model. Calculated parameters obtained via fitting of TTCF using SASview. | |


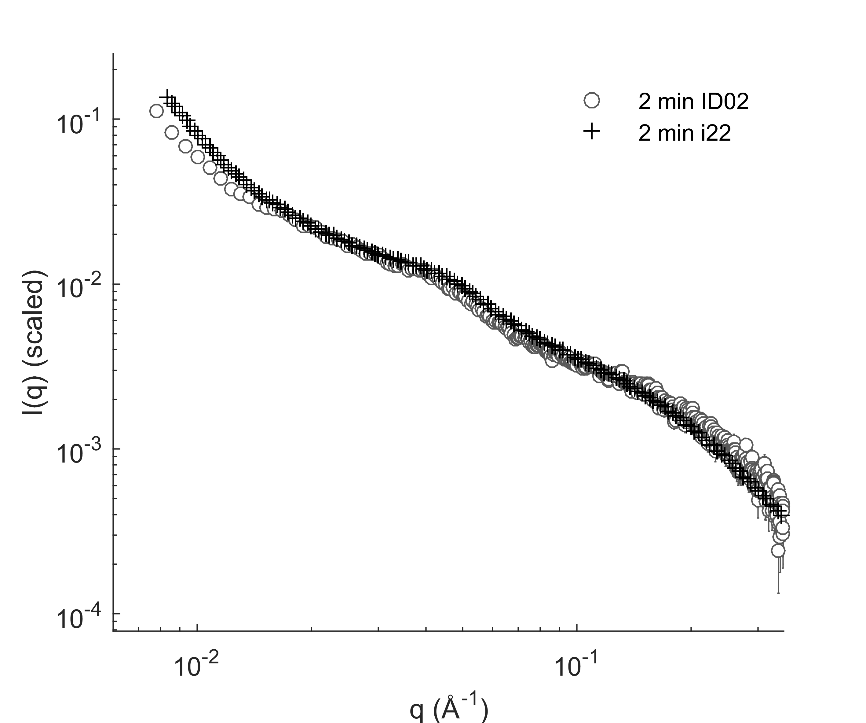


Figure S10. SAXS scattering overlap at 120 seconds **measured at i22 and ID02 beamlines. The q-range is 0.008<q<0.35 Å^-1^**

# TTCF ENSILICATION FITS

Modelling of ensilication during onset and longer evolution involved the following models and these have been designated model parameter keys, P, relating to SASview output tables described further.

## STAGE I

**i22, *in situ,* 0 - 60 seconds.** X-ray scattering measured *in situ* after addition of silica, and up to 60 seconds, was fitted with a combination of several models, each with a physical justification as follows. The power law model was intended to indicate any large surfaces or aggregated protein and silica particulates. The ellipsoidal model was used to represent the protein and the silica layer deposited immediately upon its surface as a particle. A fractal growth of silica on a larger scale is represented with a fractal model. A combination of these models, visualised below, indicated a reasonable fit with χ^2^ below 10.

(P1) power law + (P2) ellipsoid + (P3) mass fractal^12^ (stage I)
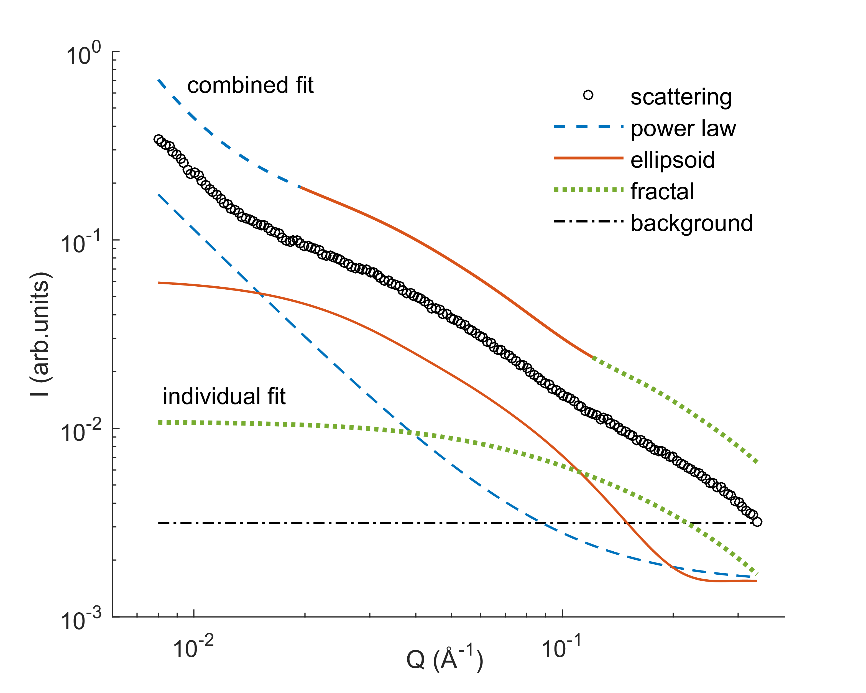

$$I\left( q \right)=\text{scale}\cdot q^{-\text{power}}+ P\left( q,\alpha\right)+scale\cdot P\left( q \right)\cdot S\left( q \right)+background$$

where: $P(q)=F(qR)^{2}$ and $S(q)=\frac{\Gamma(D_{m}-1)\zeta^{D_{m}-1}}{{[1+(q\zeta)^{2}]}^{(D_{m}-1)/2}}\frac{sin[(D_{m}-1)tan^{-1}(q\zeta)]}{q}$

with R: radius building block, D_m_: fractal dimension, $\zeta(zeta)$: cut-off length.

Where $P\left( q,\alpha\right)=\frac{\text{scale}}{V}F^{2}\left( q,\alpha\right)$ and $F(q,\alpha)=\frac{3\Delta\rho V(\sin[qr(R_{p},R_{e},\alpha)]-\cos[qr(R_{p},R_{e},\alpha)])}{[qr(R_{p},R_{e},\alpha)]^{3}}$

with R_p_: polar radius, R_e_: equatorial radius,$\Delta\rho$: density of particle, α: axis angle, $V$: ellipsoid volume; $V=(4/3)\pi R_{p}R_{e}^{2}$

## STAGE II

**i22, in situ 60 - 120 seconds and ID02, 2 - 5 mins.** From around 40 seconds onwards the fit of the Stage I model gradually worsens and by around 60 seconds the scattering has a visibly different profile, indicating that the Stage I model is no longer an appropriate description of the data. The profile is now better described by the combination of the mass fractal with a (Lorentzian) broad-peak model, which also includes a power-law component; thus the broad peak replaces the power law and ellipsoid components of the Stage I model. Broad peak scattering is typical of soft condensed matter with a complex structure having a characteristic length scale between scattering inhomogeneities. The peak of the Lorentzian, *q*_0_, is consistently found at around 0.03 Å^-1^, corresponding to a characteristic length scale of around 200 Å, broadly consistent with the typical diameter of the (protein + silica) ellipsoids of the Stage I fit. The χ^2^ values for the Stage I and Stage II fits show clearly that the broad peak model is more appropriate from 60 seconds onwards.

(P1) broad peak + (P2) mass fractal (stage II)


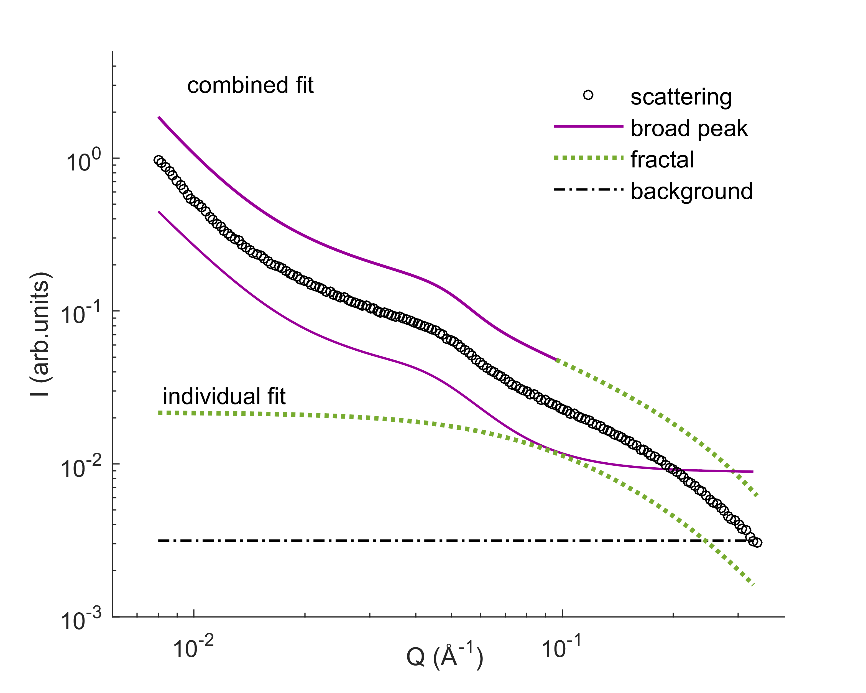

$$I\left( q \right)=\frac{A}{q^{n}}+\frac{C}{1+(|q-q_{0}|\xi)^{m}}+ scale\cdot P\left( q \right)S\left( q \right)+background$$

Where: A: Porod law scale factor, *n:* the Porod exponent, C*:* Lorentzian scale factor, *m* the exponent of *q*, ξ: the screening length.


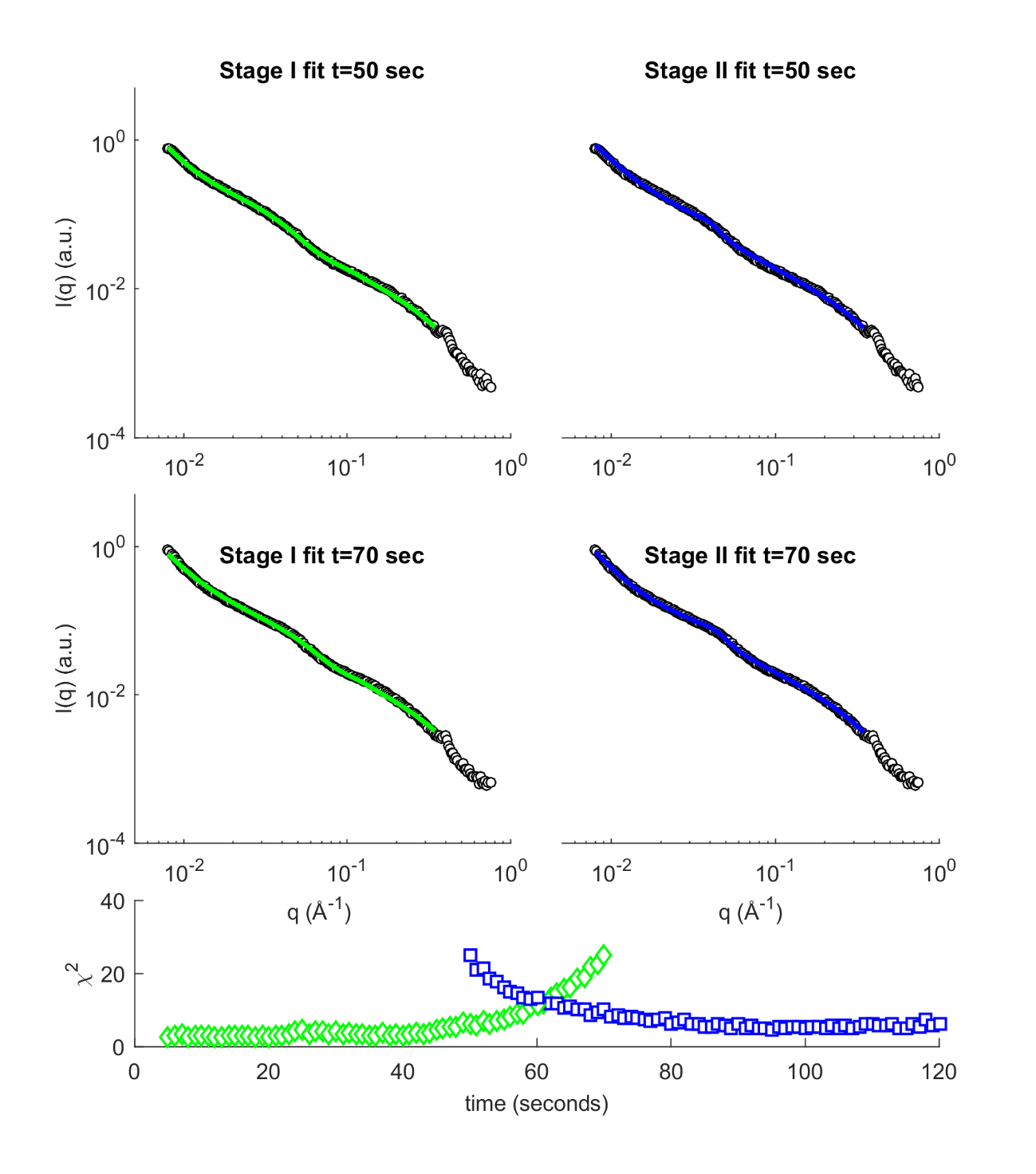


## **Figure S11. Comparative fits of the Stage I and Stage II models to data from the first two minutes of *in-situ*-initiated ensilication.**

## STAGE III

**ID02 *in situ, ex situ* initiation, 5 - 60 mins.** Scattering measured over longer duration of time was initially well fitted by the Stage II model (broad peak + mass fractal). Over several minutes the fit worsens, with a reduction in the broad peak scattering and an increase in the mass fractal scattering from high to mid-*q*. A Stage III model, consisting of the combination of power law and mass fractal, is clearly a better fit to the data from around 5 minutes onwards. Physically this is consistent with the particulates previously represented by the ellipsoid (Stage I) and broad peak (Stage II) having now lost their individual identity and been absorbed into the growth of an aggregate.


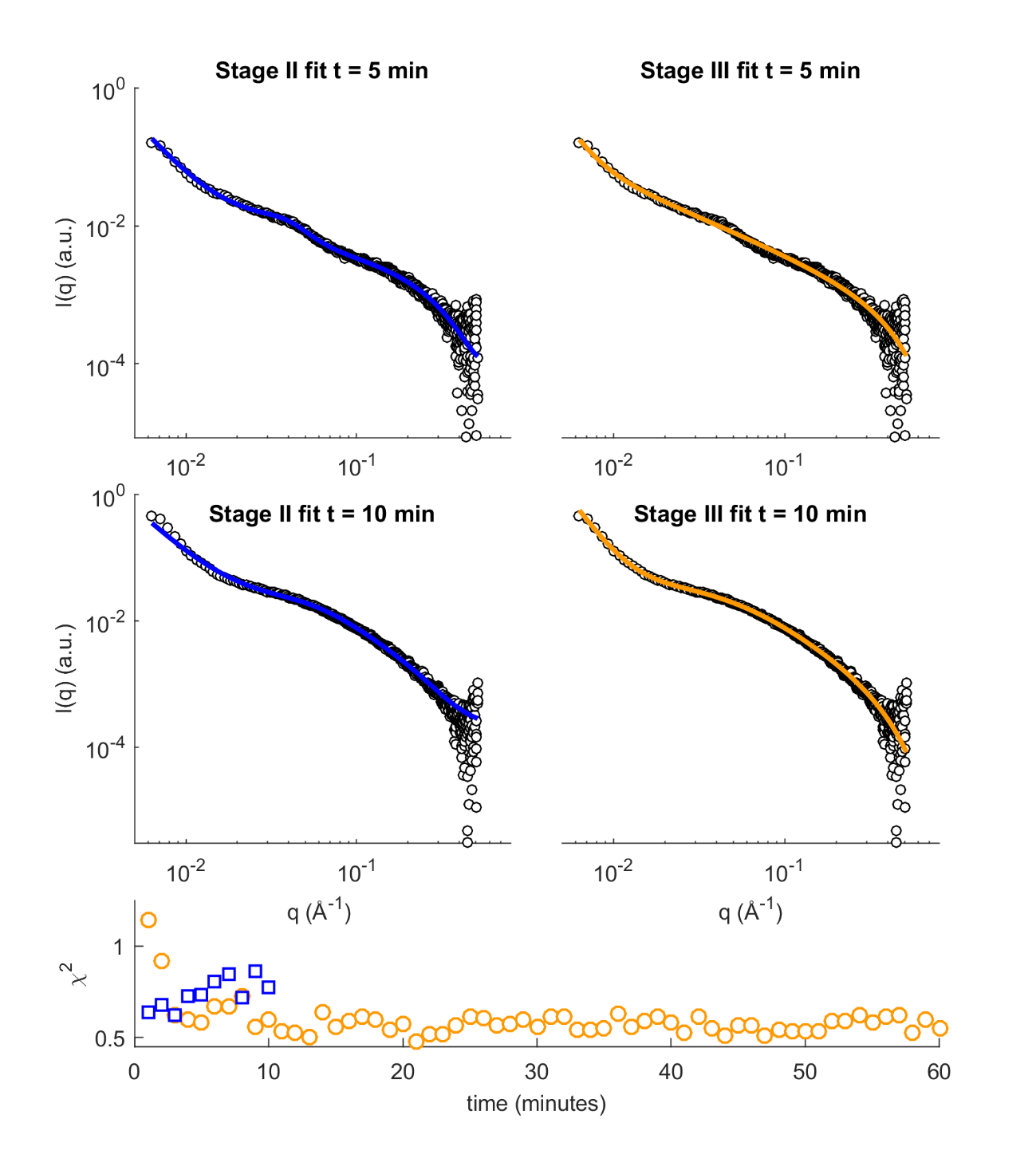


Figure S12. Comparative fits of the Stage II and Stage III models to data from *ex-situ*-initiated ensilication.

(P1) power law + (P2) mass fractal (stage III)


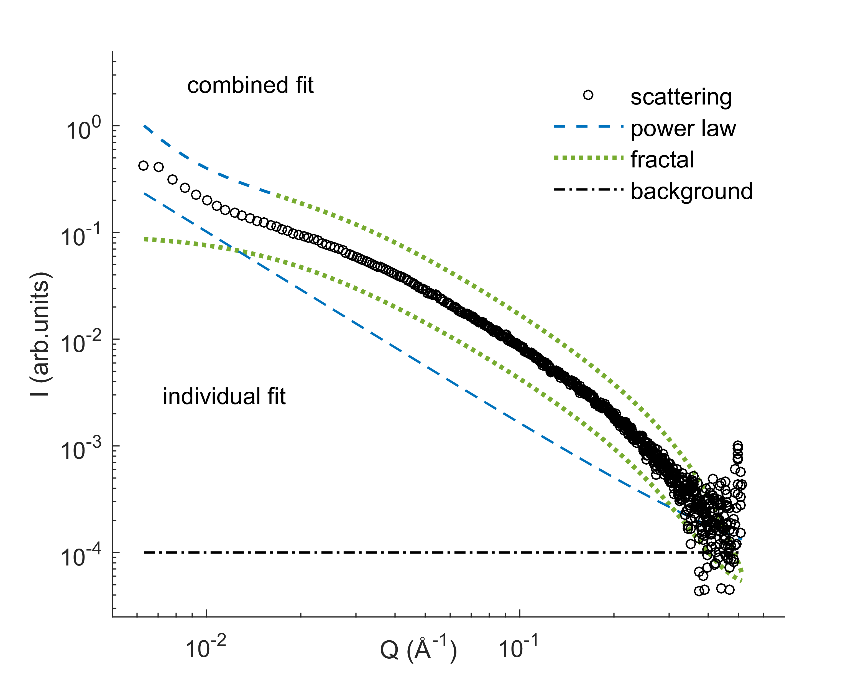

$$I\left( q \right)=\text{scale}\cdot q^{-\text{power}}+scale\cdot P\left( q \right)\cdot S\left( q \right)+background$$

Note that each model and combined models in SASview have a scalar and background value. These are accounted for when fitting 1D SAXS data.

# FIT TABLES – PARAMETER OUTPUT

# TTCF ENSILICATION SUPPLEMENTARY PLOTS

| 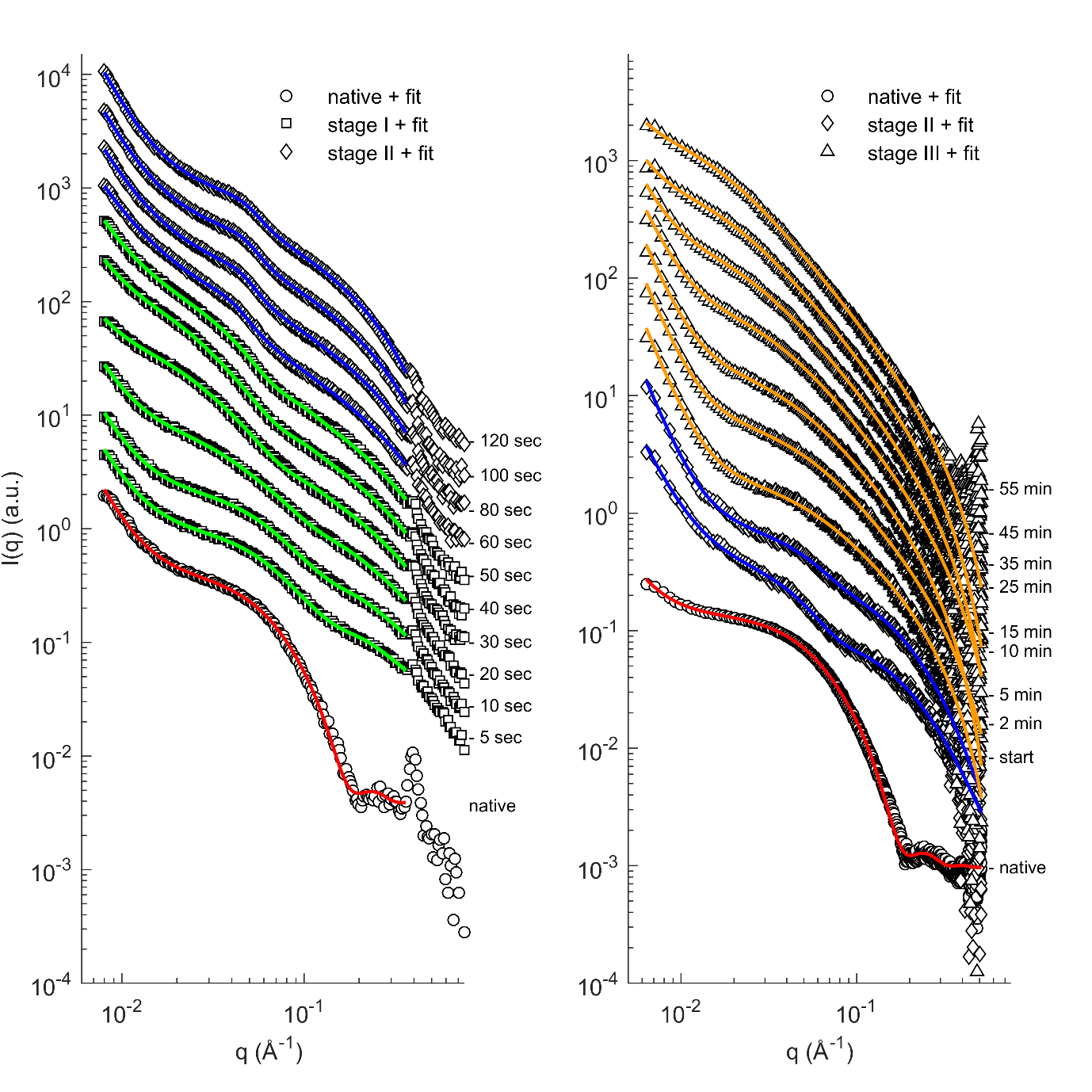 |
| --- |
| Figure S13. Full q-range of TTCF ensilication for Diamond and ESRF. (left) Onset of TTCF ensilication scattering obtained from i22. Fitting was done up to 0.35 Å^-1^ as there was Kapton interference at high q. (right) Scattering of TTCF ensilication from ID02 with full q-range fits from 0.008 – 0.51 Å^-1^. |

| 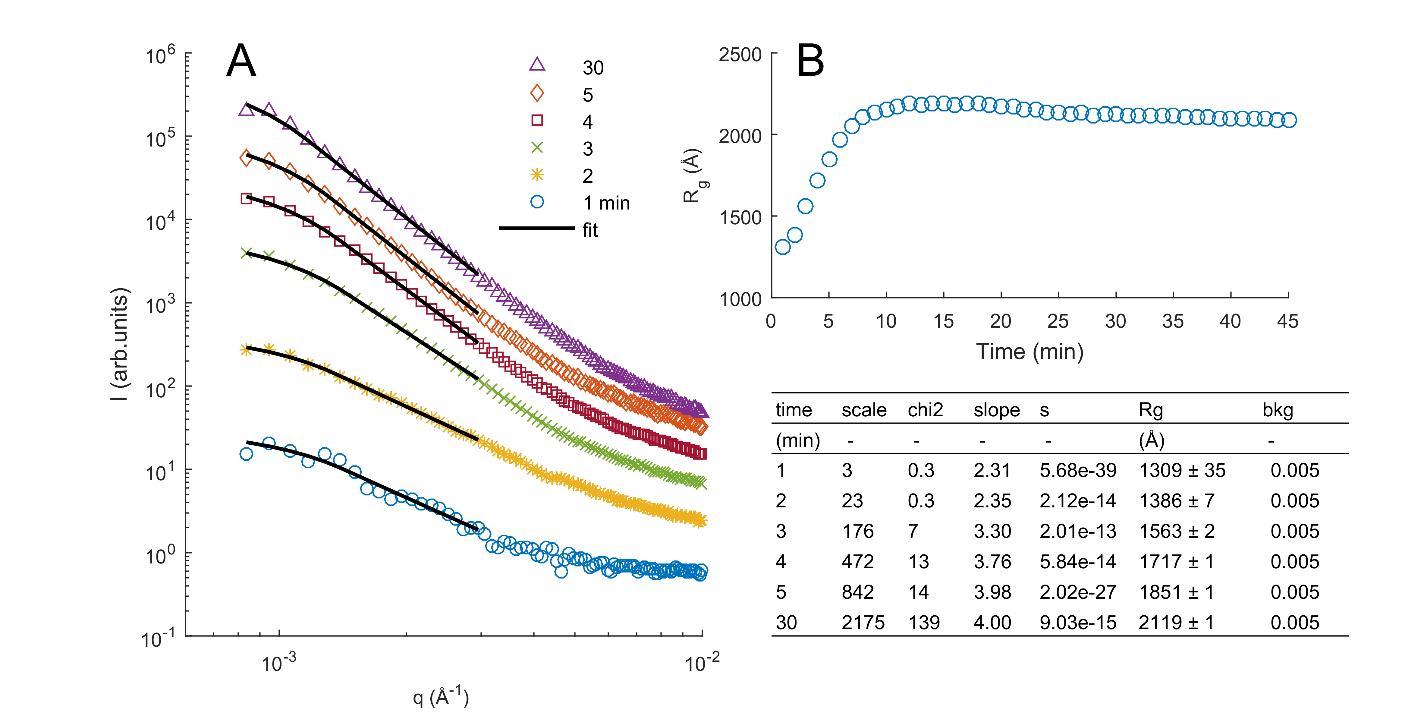 |
| --- |
| Figure S14. Guinier-Porod analysis of 0.0008<q<0.08 A-1 range SAXS 1D data. (A)Time resolved data analysis of 0.0008<q<0.08 A^-1^ of 1:50 ensilication of TTCF. Data reveals large particle aggregation initiated shortly after initiation. (B, table ) Apparent from the data is the stabilisation of particles between 2000 and 2200 Å (200-220 nm). This suggests TTCF ensilicated particles are becoming larger and subsequently the scattering intensity is increasing at lower *q* (i.e. moving out of the *q*-range) during the onset of ensilication. From this, we argue that the residual scattering observed from the data during stage II and III are purely silica. |

**References**

1 Hewitt, E. W. *et al.* Natural processing sites for human cathepsin E and cathepsin D in tetanus toxin: implications for T cell epitope generation. *The Journal of Immunology* **159**, 4693-4699 (1997).

2 Webb, B. & Sali, A. Comparative Protein Structure Modeling Using MODELLER. *Curr Protoc Bioinformatics* **54**, 5.6.1-5.6.37, doi:10.1002/cpbi.3 (2016).

3 Marti-Renom, M. A. *et al.* Comparative protein structure modeling of genes and genomes. *Annu Rev Biophys Biomol Struct* **29**, 291-325, doi:10.1146/annurev.biophys.29.1.291 (2000).

4 Knapp, M. & Segelke, B., Rupp, B. *The 1.61 Angstrom Structure of the Tetanus Toxin Ganglioside Binding Region: Solved by MAD and Mir Phase Combination*. (1998).

5 Schneidman-Duhovny, D., Hammel, M., Tainer, J. A. & Sali, A. Accurate SAXS profile computation and its assessment by contrast variation experiments. *Biophysical journal* **105**, 962-974, doi:10.1016/j.bpj.2013.07.020 (2013).

6 Schneidman-Duhovny, D., Hammel, M., Tainer, J. A. & Sali, A. FoXS, FoXSDock and MultiFoXS: Single-state and multi-state structural modeling of proteins and their complexes based on SAXS profiles. *Nucleic Acids Research* **44**, W424-429, doi:10.1093/nar/gkw389 (2016).

7 Moore, P. Small-angle scattering. Information content and error analysis. *Journal of Applied Crystallography* **13**, 168-175, doi:doi:10.1107/S002188988001179X (1980).

8 Guinier, A. & Fournet, G. *Small‐Angle Scattering of X‐rays*(John Wiley & Sons, Inc., 1955).

9 Chen, Y. C. *et al.* Thermal stability, storage and release of proteins with tailored fit in silica. *Sci Rep* **7**, 46568, doi:10.1038/srep46568 (2017).

10 Fischer, H., de Oliveira Neto, M., Napolitano, H. B., Polikarpov, I. & Craievich, A. F. Determination of the molecular weight of proteins in solution from a single small-angle X-ray scattering measurement on a relative scale. *Journal of Applied Crystallography* **43**, 101-109, doi:doi:10.1107/S0021889809043076 (2010).

11 Feigin, L. A. & Svergun, D. I. *Structure Analysis by Small-Angle X-Ray and Neutron Scattering*. ( Springer, 1987).

12 Hall, D. F. R. M. a. P. L. Small-angle scattering from porous solids with fractal geometry. *Journal of Physics D: Applied Physics* **19**, 1535 (1986).
